# Transcranial Calcium Macro‐Imaging From the Auditory Cortex of Thy1‐Cre‐Driven GCaMP8 Transgenic Rats

**DOI:** 10.1002/npr2.70113

**Published:** 2026-04-28

**Authors:** Manavu Tohmi, Hisaaki Namba, Meiko Kawamura, Ryuichi Hishida, Shota Iguchi, Kazuto Kobayashi, Natsuki Matsushita, Tomoji Mashimo, Rie Natsume, Kenji Sakimura, Hiroyuki Nawa

**Affiliations:** ^1^ Department of Physiological Sciences School of Pharmaceutical Sciences, Wakayama Medical University Wakayama Japan; ^2^ Department of Animal Model Development Brain Research Institute, Niigata University Niigata Japan; ^3^ Department of Neuroscience of Disease Brain Research Institute, Niigata University Niigata Japan; ^4^ Department of Molecular Genetics Institute of Biomedical Sciences, Fukushima Medical University School of Medicine Fukushima Japan; ^5^ Division of Laboratory Animal Research Aichi Medical University School of Medicine Aichi Japan; ^6^ Division of Animal Genetics Laboratory Animal Research Center, Institute of Medical Science, the University of Tokyo Tokyo Japan

**Keywords:** auditory cortex, functional mapping, GCaMP, Thy1, transgenic rat

## Abstract

**Background:**

Genetically encoded calcium indicators such as GCaMP enable us to monitor neuronal activities from living animals at synaptic levels as well as at whole‐brain levels. However, the genetic variation of GCaMP transgenic rats is limited compared with the various demands for neuroscience studies on rats.

**Method:**

We established two transgenic rat lines: one expressing Cre recombinase under the control of the Thy1 promoter and another knock‐in line carrying a floxed STOP cassette followed by GCaMP8 inserted into the β‐actin locus. We mated these two transgenic rats and produced the Thy1‐Cre‐driven GCaMP8‐expressing rats.

**Results:**

Approximately 80% of cortical neurons were positive for the GCaMP immunoreactivity. The GCaMP8‐expressing rats were subjected to transcranial calcium imaging from the auditory cortex under anesthesia. With this simple procedure, we were able to detect the maximum responses to 68 dB click sounds;ΔF/F_0_ = 1.2%. The rise time reaching the peak response was approximately 150 ms, and the half decay time was about 150 ms.

**Conclusions:**

These results suggest that the present GCaMP8‐expressing rats as well as their parental transgenic rat strains offer a new tool for cortical macro‐imaging and functional mapping with the transcranial approach.

## Abstract 2

We generated Thy1‐Cre–driven GCaMP8 transgenic rats by mating Thy1‐Cre rats with knock‐in rats carrying a floxed GCaMP8‐STOP cassette in the β‐actin locus. Robust neuronal GCaMP8 expression enabled transcranial calcium imaging of the auditory cortex, establishing this model as a powerful tool for minimally invasive cortical macro‐imaging and functional mapping.

## Graphical abstract

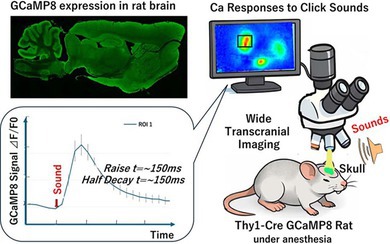

## 1 Introduction

Mice have been extensively used in the production of genetically modified animals because of their cost‐effectiveness and the minimal space required for their breeding. Historically, embryonic stem (ES) cells, essential for creating genetically modified animals, were established much earlier in mice than in rats. Consequently, the number of genetically modified mice registered in public databases is around 15 000 [1], whereas the number of genetically modified rats is estimated to be between 1000 and 2000 according to the information of the world's largest rat strain repository, the Rat Resource & Research Center (RRRC) [2]. However, with the establishment of rat ES cells and the availability of the CRISPR‐Cas9 system, the technical challenges of using rats have been nearly overcome [3]. Nonetheless, issues related to the cost and space required for rat housing remain unresolved, which is why the number of genetically modified rats is increasing slowly.

Fundamentally, mice, with their smaller brains, are advantageous for monitoring fine neural structures and synaptic activity, as they do not require larger‐sized experimental equipment [4]. However, the small size of the mouse brain makes it difficult to manipulate or target specific regions, making rats—with their larger brains—more suitable for studies requiring precise localization or imaging of the small regions. Another significant difference between mice and rats is their cognitive and behavioral abilities [5]. Rats are capable of handling more complex learning tasks, such as the 5‐choice serial reaction time task [5, 6]. Over more than 20 years of schizophrenia model research, we have frequently used rats in experiments such as vocalization modeling, electroencephalogram (EEG) studies, and complex cognitive‐behavioral tests, taking advantage of their superior cognitive abilities and larger brain size [7]. Accordingly, we aimed here to establish GCaMP‐transgenic rats for macro‐calcium imaging from the brain.

Among the various calcium indicators [8, 9, 10, 11, 12], we selected GCaMP8 because it is one of the latest GCaMP derivatives, offering an improved dynamic range (Fmax/Fmin = 38) and rapid decay (*t*_*1/2*_ = 400 ms) [13]. To achieve the cell‐specific expression of GCaMP protein, we employed the Cre‐loxP system and designed a GCaMP8 expression vector containing a loxP‐flanked STOP cassette. To remove the STOP cassette and enable GCaMP8 expression in excitatory neurons, we generated a new transgenic rat line constitutively expressing Cre recombinase under the control of the mouse Thy1.2 promoter. Crossing these rat lines produced offspring that express GCaMP8 in the brain. Using these GCaMP8 transgenic offspring, we monitored neocortical calcium signals transcranially with a minimally invasive surgical procedure [14]. We focused on the auditory cortex, as previous studies employing GCaMP‐based macro‐imaging in the auditory cortex were done in mice and relatively limited in rats [15, 16]. In addition, two‐photon microimaging in this region has often relied on viral vector–mediated GCaMP expression [17, 18, 19, 20]. In this study, therefore, we discuss the advantages and limitations of transcranial macro‐imaging using GCaMP8‐expressing transgenic rats.

## 2 Experimental Methods

### 2.1 Animals

Rats were housed with 2–3 per cage in the Niigata University Animal Facility and in the Wakayama Medical University Animal Facility under a 12‐h light/dark cycle (8:00 am ON and 20:00 pm OFF) at constant temperature and humidity. Solid food and water were available *ad libitum*. All animal experiments were approved by the Animal Care and Use Committee of Niigata University and Wakayama Medical University, and in accordance with the Guide for the Care and Use of Laboratory Animals of the Japan Neuroscience Society.

### 2.2 Establishment of Thy1.2‐Promoter‐Driven Cre Transgenic Rats

**FIGURE 1:**
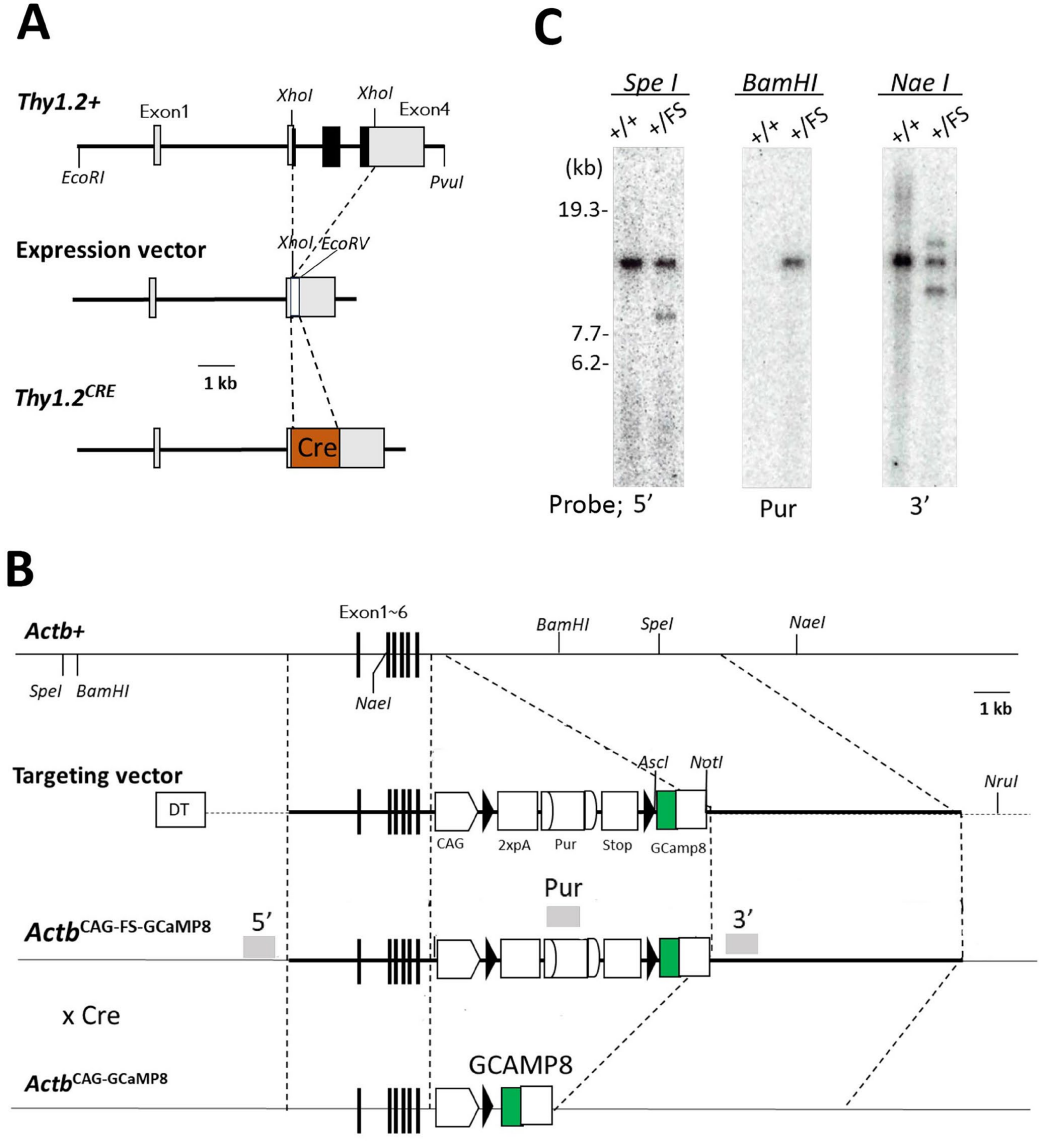
Transgene construction and Cre‐loxP recombination. (A) A Cre recombinase gene was inserted into a Thy1 promoter–driven expression vector, located between exons 2 and 4 of the murine *Thy1.2* gene. The resulting DNA construct was injected into fertilized rat eggs for genomic integration. (B) The knock‐in vector was generated by adding homologous arm regions of the rat *β‐actin* gene plus *diphtheria toxin* gene (DT) and its 3′ non‐coding region to a cassette containing the CAG promoter, the floxed STOP cassette [the double polyadenylation signals (2xpA), FRT‐flanked puromycin‐resistance domain (Pur), and transcription termination domain (STOP)] and the GCaMP8 gene. (C) This construction was transduced into rat ES cells, and homologous recombinants were identified by Southern blotting. The recombination‐confirmed ES cells (FS) were injected into rat blastocysts. The resulting floxed GCaMP8 knock‐in rats were crossed with Thy1 promoter–driven Cre transgenic rats to excise the STOP cassette and activate GCaMP8 expression. The locations of the three DNA probes used in Southern blotting are indicated by gray boxes labeled 3′, Pur, and 5.

The expression vector was obtained from Addgene (#20736). It contains a 6.5‐kb fragment of the murine Thy1.2 gene, extending from the promoter through the intron following exon 4, but lacking exon 3 and its flanking introns (Figure 1). The pBS185 plasmid, containing cytomegalovirus (CMV) promoter and Cre recombinase sequence, was obtained from Addgene (Plasmid #11916), digested with MluI. The resulting DNA ends were blunted and then digested with XhoI. After gel purification, the DNA fragment of Cre recombinase was inserted into the EcoRV and XhoI sites of the above expression vector. The resulting construction was subjected to DNA sequencing, followed by enzyme digestion with EcoRI and AatII to obtain the complete insert of both *Thy1.2* promoter and Cre sequence. Following gel purification, the *Thy1.2* promoter–Cre cDNA fragment was microinjected into 60 fertilized Sprague–Dawley (SD) rat eggs. These eggs were implanted into a pseudopregnant SD rat uterus, resulting in live offspring. Thirty‐three pups were born, and PCR analysis identified two rats carrying the *Thy1.2* promoter and the Cre cDNA. One of these, designated line 19, carried a single copy of Cre and was used for subsequent Cre–loxP recombination experiments. Transgenic rat line 19 exhibited no apparent abnormalities in development or reproduction (data not shown).

### 2.3 Establishment of Floxed GCaMP8 Knock‐In Rats

We inserted a cassette driven by the CAG promoter around 500 bp downstream of the *Actb* gene in rat ES cells. In mice, stable expression is observed when a foreign gene is inserted into the region corresponding to this location [13]. The vector was constructed as follows. A 2.5‐kb DNA fragment containing GCaMP8 and *WPRE‐pA* [13] was subcloned into the AscI/NotI sites of a middle‐entry plasmid (pDME‐1r) containing the following elements: cytomegalovirus enhancer/chicken β‐actin (CAG) promoter, the first loxP sequence, two polyA sequences, an FRT‐flanked *hEF1α* promoter–puromycin resistance gene–polyA, the second loxP sequence, and the AscI/NotI cloning site. The DNA segment flanked by loxP sequences was referred to as a STOP cassette (FS), as described previously [21]. The 5′ homology arm (4.7 kb) and 3′ homology arm (8.74 kb) were retrieved from a rat bacterial artificial chromosome (BAC) clone (CHORI‐230‐371‐F9) and inserted into the 5′ entry plasmid (pD5UE‐2) and the 3′ entry plasmid (pD3DE‐2), respectively, using the Quick and Easy BAC Modification Kit (Gene Bridges, Dresden, Germany) [22].

For targeting vector assembly, the above entry plasmids were recombined into a destination vector plasmid (pDEST‐DT, containing a CAG promoter–driven diphtheria toxin gene) using the MultiSite Gateway Three‐Fragment Vector Construction Kit (Invitrogen, Thermo Fisher Scientific, MA, USA). The targeting vector, linearized with NruI, was electroporated into rat embryonic stem (ES) cells (LH003), which had been originally established from the Lister Hooded strain [23]. ES cell clones carrying the targeted homologous recombination were identified by Southern blot analysis and injected into 120 rat blastocysts (SD strain) to generate chimeric embryos. Chimeric rats exhibiting the Lister Hooded coat color were backcrossed with wild SD rats to confirm germline transmission and to establish the Actb:CAG‐FS‐GCaMP8 rat line. Their offspring were further backcrossed 7 times with wild SD rats. Genomic DNA was extracted from auricular tissue, and genotyping was performed by PCR using the following primers: F1, 5′‐TGTACACTGACGTGAGACCGTTT‐3′; R1, 5′‐AGGCAGGTGCCTATCTGGTT‐3′; and R2, 5′‐GATGGGGAGAGTGAAGCAGAACGT‐3′. Male homozygous Actb:CAG‐FS‐GCaMP8 rats were mated with female Thy1.2‐driven Cre transgenic rats. The resultant Cre‐loxP recombination, in which a 4019‐bp STOP cassette flanked by loxP sites was excised, was confirmed by PCR using the following primers: F2, 5′‐GGCTCTAGAGCCTCTGCTAA‐3′ and R3, 5′‐AGAAGTCGTGCTGCTTCATG‐3′.

### 2.4 Immunohistochemistry

Rats (postnatal days 75–90) were deeply anesthetized with a combination anesthetic containing 0.375‐mg/kg medetomidine (Kyoritsu Seiyaku), 2‐mg/kg midazolam (Sandoz), and 2.5‐mg/kg butorphanol (Meiji Seika Pharma) via intraperitoneal injection and then perfused transcardially with room‐temperature PBS followed by buffered 4% paraformaldehyde (PFA). The brain was harvested and post‐fixed in 4% PFA overnight at 4°C, washed with PBS, and sectioned coronally and sagittally at 25 μm thickness using a cryostat. Sections were incubated with blocking buffer (5% normal goat serum, 0.2% Triton X‐100 in PBS) for 1 h at room temperature. Sections were then incubated overnight at room temperature with rabbit anti‐GFP antibody (Molecular Probes, A11122; 1:300) for GCaMP detection; selected slices were co‐incubated with mouse anti‐NeuN antibody (Millipore, MAB377; 1:500) or with mouse anti‐glutamate decarboxylase 65 (GAD65) antibody (Sigma #5111; 1:500). After incubation with primary antibodies, sections were washed three times in PBS (20 min each) and incubated for 2 h at room temperature with Alexa 488–conjugated goat anti‐rabbit IgG and Alexa 555–conjugated goat anti‐mouse IgG secondary antibodies (both Invitrogen; 1:200). Sections were washed in PBS, mounted onto slides with 100% glycerol, and imaged using a Keyence All‐in‐One fluorescence microscope equipped with 4×, 10×, and 20× objectives.

### 2.5 Auditory Stimulation

A click‐shaped band noise stimulus was used to evoke fluorescent signals of GCaMP8 in the auditory cortex. The band noise, spanning frequencies from 1.25 to 40 kHz, had a click‐shaped waveform with a 12.5 μs rise time and an exponential decay (half‐life: 3 ms) extending to a total duration of 50 ms. Peak sound pressure levels (SPL) ranged from 42 to 90 dB SPL in 2 dB increments. Auditory stimuli were played using a digital music player (DX180, iBasso Audio, China) with a sampling rate of 384 kHz, and delivered from a speaker (TSE‐1010, Pioneer, Japan) positioned 30 cm in front of the rat. Inter‐sound intervals were randomized between 250 and 1750 ms, and the order of sound presentations was randomized. Each sound condition was presented 80 times to eliminate spontaneous background calcium signals by averaging.

### 2.6 Transcranial Fluorescence Imaging

Rats (postnatal days 35–50) were anesthetized with urethane (1.6 g/kg body weight, intraperitoneally), and rectal temperature was maintained at 37.5°C using a heating pad. In the present study, urethane anesthesia was selected because it provides a long‐lasting and stable anesthetic state with minimal effects on excitatory synaptic transmission and thalamocortical firing, particularly in the auditory system [24, 25]. Given the carcinogenic properties of urethane in animals, all rats were euthanized with isoflurane gas immediately following transcranial imaging.

The scalp was removed, and a metal holder was affixed to the skull with dental resin. The temporal muscle over the auditory cortex was removed, and the skull surface was cleaned and covered with mineral oil to maintain optical clarity and reduce light scattering. The skull was positioned under a fluorescence microscope (DFLFPSP2R, Brainvision, Japan). GCaMP8 fluorescence was excited using a 470 nm LED light source (M470L5, Thorlabs, USA) and collected through a 500–550 nm band‐pass filter. Images (240 × 135 pixels, field size 7.1 × 4.0 mm) of the GCaMP signal from the brain surface were recorded at a sampling rate of 20 Hz through the temporal bone using a CCD camera (BU‐61 M, Bitran, Japan) mounted on the microscope. In the image processing procedure, images were averaged across all trials. The ratio of each image (ΔF/F_0_) at each time point or of the 2nd–4th post‐stimulus frames to the baseline image obtained by averaging the three pre‐stimulus frames was then calculated [26]. The stimulation and data acquisition system—including CCD camera control, the sound trigger signals, and the image‐processing interface—was all operated using a self‐developed LabVIEW program (National Instruments LTD) (Data S2: Supplement # 11, with README). Pixel values within the region of interest (ROI) defined on each image were averaged to eliminate event‐unrelated background activity and were used as the data sets for figure drawing and statistical analysis (Data S3: Supplement # 12, with README).

### 2.7 Statistical Procedures

Fluorescent responses of ΔF/F_0_ were subjected to statistical analysis of planned *t*‐test with or without Holm's compensation. Values are represented as mean ± SE. *P* less than 0.05 was considered significant.

## 3 Results

### 3.1 Establishment of Thy1 Promoter‐Driven Cre Transgenic Rats

Because murine Thy1 is predominantly expressed in projection neurons, its promoter region—including exon 1, intron 1, and exon 2—has been widely used to drive exogenous gene expression in rodent neurons [16]. In this study, we employed the Thy1.2 promoter to achieve neuron‐specific expression of Cre recombinase. The transgene construct, comprising the Thy1 promoter, the Cre recombinase coding sequence, and the polyadenylation domain of the Thy1 gene (Figure 1A), was microinjected into 256 fertilized Sprague–Dawley rat eggs, yielding 89 offspring. Transgenic rat lines were identified by PCR analysis of tail DNA using multiple primer sets (Data S1: Supplement #1). Two animals carried the transgene, and one was confirmed to harbor a single copy of the transgene and to express enzymatically active Cre recombinase in the brain (data not shown).

### 3.2 Characterization of GCaMP8 Expression in the Brain of the Offspring From Floxed GCaMP8 Knock‐In Rats Mated With Thy1 Promoter‐Driven Cre Rats

We employed a knock‐in strategy in a rat ES cell line to establish floxed knock‐in GCaMP8 rats. The targeting vector was designed to contain the CAG promoter, double polyadenylation signals, an FRT‐flanked puromycin‐resistance domain, a transcription termination domain, the GCaMP8 cDNA, an RNA‐stabilizing sequence (WPRE), and a polyadenylation signal. The DNA region containing the double polyadenylation signals, FRT‐flanked puromycin‐resistance domain, and transcription termination domain was flanked by loxP sites and referred to as a STOP cassette (Figure 1B). This GCaMP8 expression vector was transfected into the rat ES cell line, yielding 35 puromycin‐resistant colonies [23]. We performed Southern blotting using three DNA probes (Figure 1B) and identified ES cell clones containing correct knock‐in integration of the STOP cassette and GCaMP8 (Figure 1C). Properly targeted ES cells were injected into 120 rat blastocysts, resulting in five viable transgenic rat lines. These lines were subsequently backcrossed to the Sprague–Dawley strain for more than seven generations.

**FIGURE 2:**
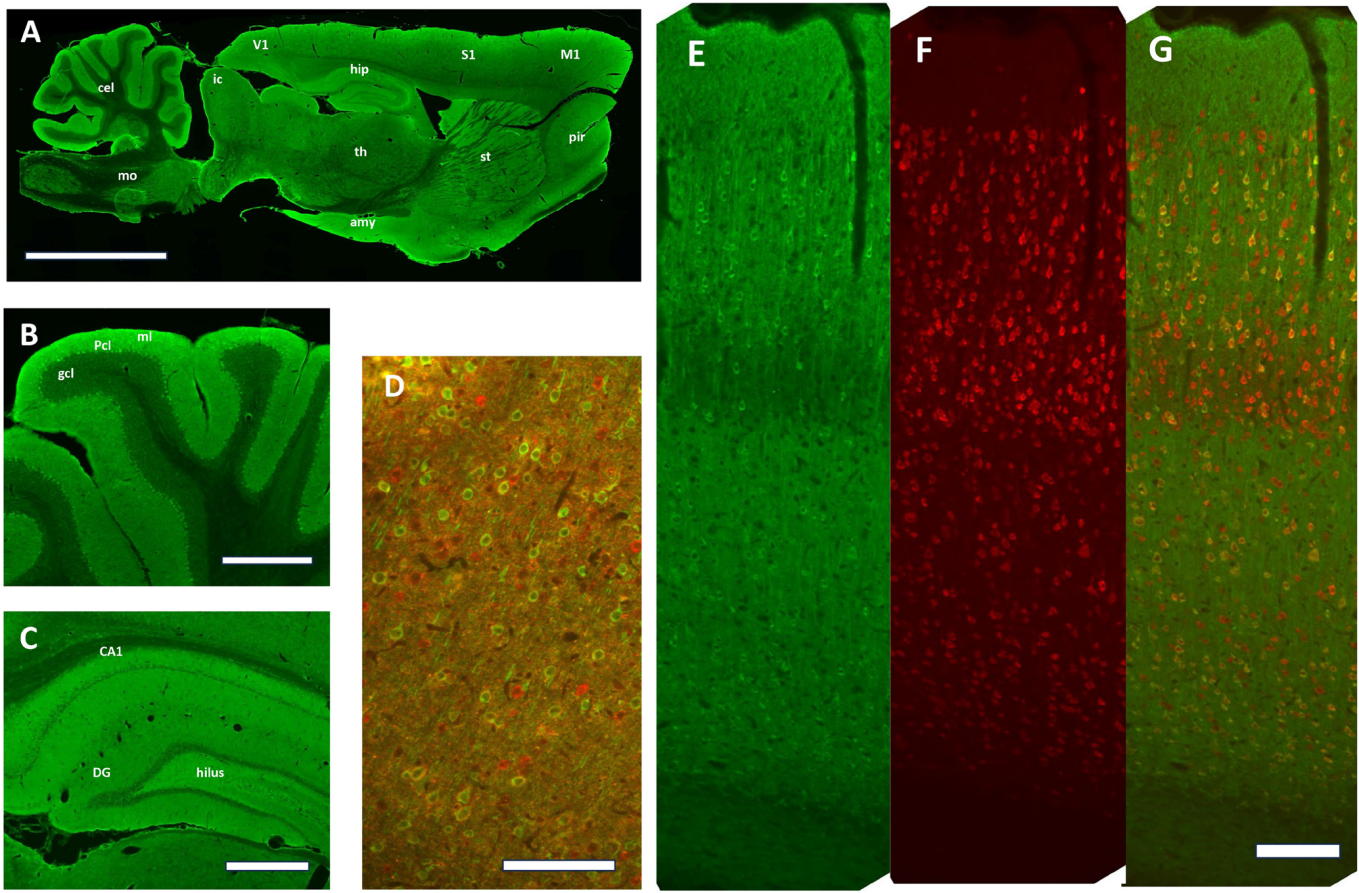
Distribution of GCaMP‐expressing cells in the brain of Thy‐1‐Cre‐driven GCaMP8‐expressing rats. The distribution and cellular localization of GCaMP8 protein were examined by immunostaining with the antibodies against GFP (for GCaMP8), glutamate decarboxylase 67 (GAD) (for GABAergic neurons), and NeuN (for neuronal cells). (A) Whole‐brain distribution of GCaMP immunoreactivity (green) in the sagittal section. (B) Cerebellar distribution of GCaMP immunoreactivity (green). (C) Hippocampal distribution of GCaMP immunoreactivity (green). (D) Immunoreactivities for GCaMP (green) and GAD67 (red) in the layers 2/3/4 of the auditory cortex. (E) Immunoreactivities for GCaMP (green) in the whole layers of the auditory cortex. (F) Immunoreactivities for NeuN (red) in the whole layers of the auditory cortex. (G) Color merged image of panel E and panel F in the same field. Abbreviations: mo; medulla oblongata, cel; cerebellum, Ic; inferior colliculus, Hip; hippocampus, V1; primary visual cortex, S1; Somatosensory cortex, M1; Primary motor cortex, Pir; piriform cortex, St; striatum, th; thalamic nucleus, Amy; amygdala, ml; molecular layer, Pcl; Purkinje cell layer, gcl; granule cell layer. Scales bars; 5 mm in A, 1 mm in B and C, 100 μm in D–G. The original, non–contrast‐adjusted and non‐cropped raw TIFF images are provided as Data S1: Supplement #2–#10.

Floxed GCaMP8 knock‐in rats were mated with the Thy1 promoter–driven Cre transgenic rats described above to generate GCaMP8‐expressing knock‐in rats (Figure 1). Using offspring in which the STOP cassette had been excised, we examined the expression and distribution of GCaMP8 in the brain. Sagittal and coronal brain sections were subjected to immunostaining with antibodies against GFP to visualize GCaMP8 expression (Figure 2) (Data S1: Supplement #2–#10). High levels of GFP immunoreactivity were observed in the cerebellum, hippocampus, upper layers of the neocortex, and amygdala (Figure 2A). In the cerebellum, strong GFP immunoreactivity was detected in the Purkinje cell layer and molecular layer (Figure 2B). In the hippocampus, GFP immunoreactivity was modest or absent in the pyramidal cell layer, whereas strong signals were observed in dendritic regions (Figure 2C).

Coronal sections containing the auditory cortex were subjected to double immunostaining using antibodies against GFP (to detect GCaMP8) in combination with antibodies against NeuN (to label neurons) or against GAD65 (to label GABAergic neurons) (Figure 2D–G). Approximately 84% ± 5% of NeuN‐positive neurons were immunoreactive for GFP, whereas only 1.6% ± 0.4% of GFP‐positive cells lacked NeuN immunoreactivity in the auditory cortex (*n* = 5 sections from 3 rats) (Figure 2E–G). These GFP‐positive cells were predominantly excitatory neurons, as 95% ± 3.1% of GFP‐positive neurons were negative for GAD65 immunoreactivity (*n* = 2 sections from 2 rats) (Figure 2D). Together, these results indicate that a substantial proportion of excitatory neurons in the auditory cortex express GCaMP8 in Thy1‐Cre–driven GCaMP8‐expressing rats.

### 3.3 Transcranial Macro‐Calcium Imaging From the Auditory Cortex

**FIGURE 3:**
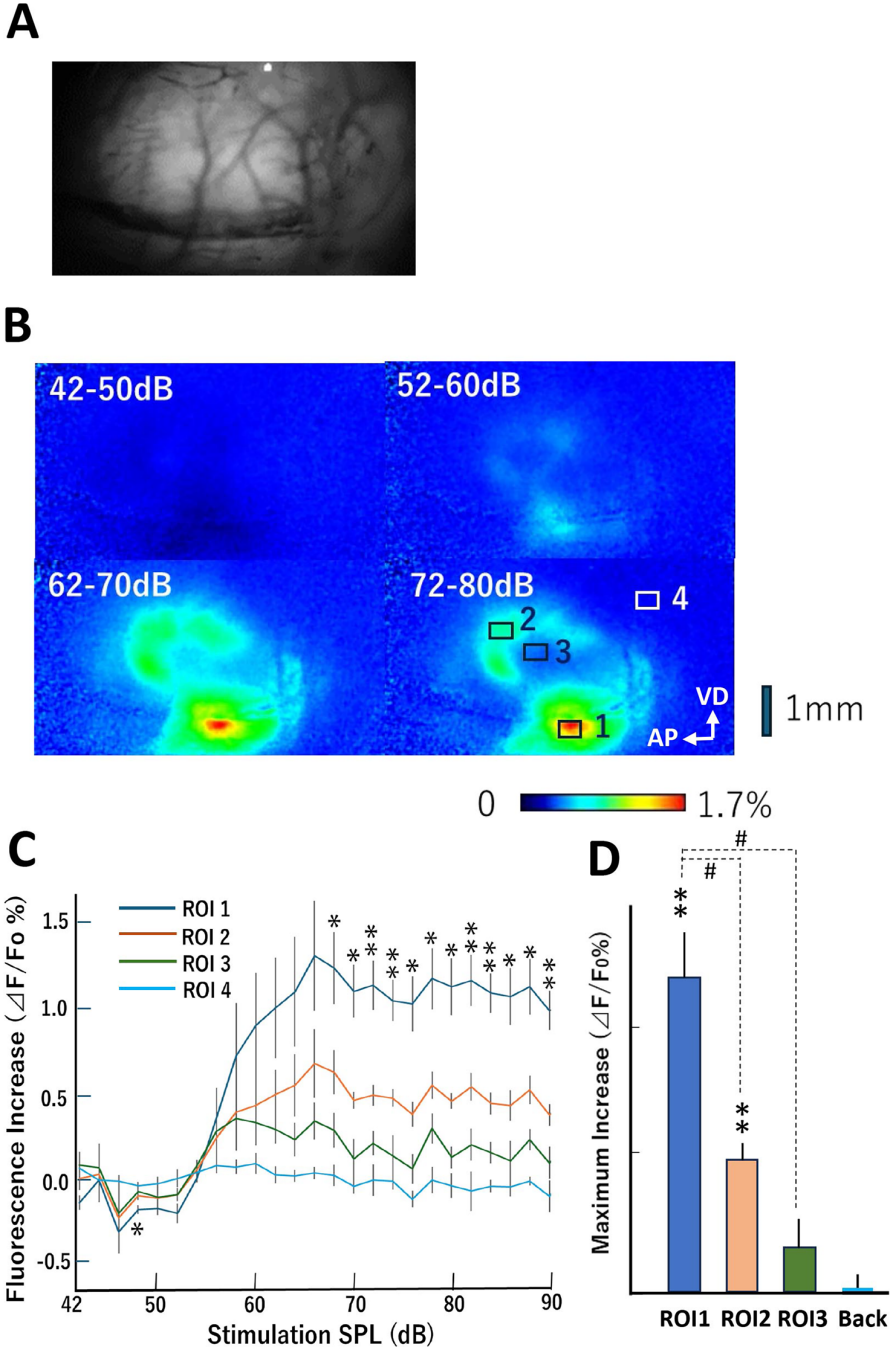
Dynamic range of sound‐evoked calcium responses in the auditory cortex. (A) Transcranial fluorescence of the right temporal cortex in Thy1‐Cre‐driven GCaMP8‐expressing rats in the absence of stimulation. (B) Thy1‐Cre‐driven GCaMP8‐expressing rats were exposed to pulse sounds of varying intensities ranging from 42 to 80 dB in 2‐dB increments. Representative averaged images from one rat are shown, obtained by averaging 80 frames for each pulse trial and further pooling every five neighboring intensity levels (i.e., 42–50 dB, 52–60 dB, 62–70 dB, and 72–80 dB) to eliminate background signals and noises, particularly at lower sound intensities. (C) The ratio of ΔF/F₀ (%) was calculated at four regions of interest (ROIs) indicated in Figure 3B and plotted against sound pressure level (SPL) (*n* = 6 rats). ROI1, ROI2, ROI3, and ROI4 were set in the suprarhinal auditory field (SRAF), the posterior auditory field (PAF), and the ventral auditory field (VAF), and background (Back), respectively. (D) Regional difference in the maximum responses to the click sound (70 dB) Statistical analyses were done by planned *t*‐test with Holm's compensation; *p < 0.05, **p < 0.01, compared with the background levels, and ^*#*^ *p < 0.05*, compared with ROI1 (A2). Abbreviations: AP, anterior–posterior axis; VD, ventral–dorsal axis. The reader‐interoperable image datasets, formatted as multipage TIFFs, are included in Data S3: Supplement #12 for each animal at every SPL. Event‐related responses corresponding to 50–100, 100–150, and 150–200 ms post‐stimulation were pooled, averaged and plotted to display the SPL dependence.

Taking full advantage of the calcium indicator, GCaMP8, we attempted to monitor its fluorescence signals transcranially through the temporal skull under anesthesia. Increasing the sound pressure levels (SPL), the GCaMP8‐expressing rats were exposed to the various strengths of click‐shaped band noises (Figure 3). Significant levels of background fluorescence of GCaMP8 were detected in the absence of stimulation (Figure 3A). The highest calcium signals were observed at ROI1 in the suprarhinal auditory field (SRAF) (Figure 3B). The minimum SPL which elicited the significant calcium signals through the skull was obtained with 56 dB SPL (*p* = 0.046, *t*‐test). The maximum response of Δ F/F_0_ was obtained at 68 dB SPL with statistical significance compared with the background (*p* = 0.034, *t*‐test with Holm's compensation) (Figure 3C). The region most sensitive to click stimuli was localized in the suprarhinal auditory field of the auditory cortex (Figure 3D).

**FIGURE 4:**
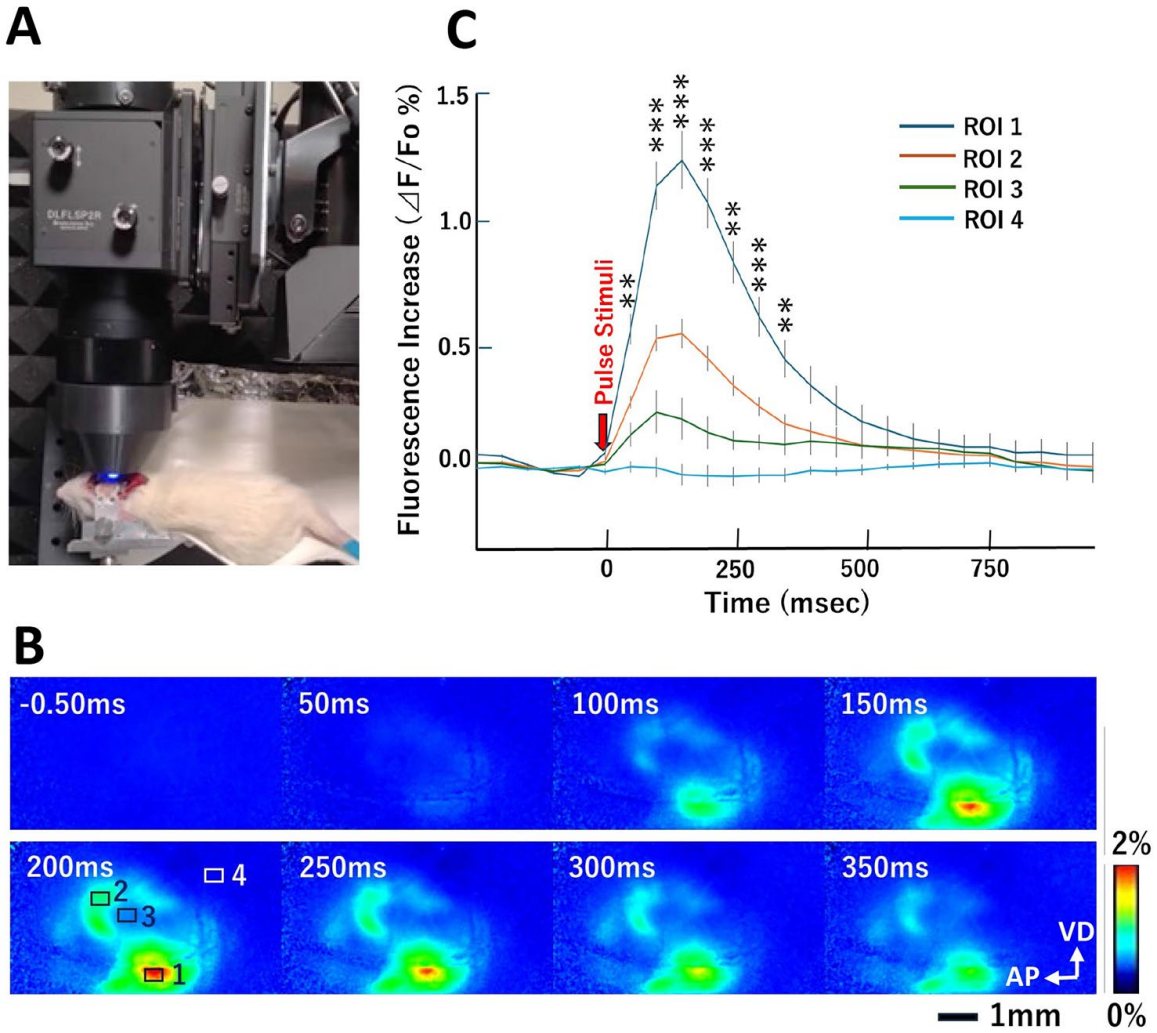
Time course of sound‐evoked calcium responses in the auditory cortex. (A) The right cortical surface of Thy1 promoter–driven GCaMP8 rats was imaged through the temporal skull using a fluorescence macroscope. Under anesthesia, rats were exposed to pulse sounds at intensities of 70–90 dB, which all elicited maximal plateau responses (see Figure 3). (B) Representative ratio images were generated every 50 ms, averaging 11 sound pressure levels (SPL) of 70–90 dB (*n* = 1 rat). (C) The ratio of ΔF/F₀ (%) was calculated every 50 ms before and after sound stimulation at four ROIs and plotted over time (*n* = 6 rats). The positions of ROIs were similarly set as described in Figure 3. Reader‐interoperable image datasets were saved in multipage TIFF format with timestamps and are provided as Data S3: Supplement #12 for each animal and SPL. Event‐related responses to 70–90 dB SPL stimuli were pooled, averaged and plotted. Abbreviations: AP, anterior–posterior axis; VD, ventral–dorsal axis. Fluorescent signal intensities at ROI1 were statistically compared with those at ROI4 (background) using planned *t‐test with Holm's compensation*; **p < 0.01, ***p < 0.001.

Next, we examined the temporal resolution of the maximal GCaMP8 fluorescence response to sound stimuli under anesthesia (Figure 4A). Calcium responses were detected as early as 50 ms after stimulus onset (Figure 4B). The rise time of GCaMP8 fluorescence following click stimuli to reach the peak was approximately 150 ms (Figure 4C). We monitored the subsequent decrease in GCaMP8 fluorescence and estimated the half decay time (*t*_1/2_) to be approximately 150 ms after the peak time, taking into account the limited temporal resolution of our CCD camera. Accordingly, these kinetic values of GCaMP8 fluorescence are roughly consistent with those reported in the original study [13].

## 4 Discussion

In the present study, we demonstrate that Thy1‐Cre–driven GCaMP8‐expressing rats enable transcranial monitoring of calcium‐dependent fluorescence signals from excitatory neurons in the auditory cortex. Notably, robust GCaMP8 expression in the superficial cortical layers was advantageous for transcranial imaging. Given the widespread distribution of GCaMP8 across the neocortex, cerebellum, and hippocampus, these GCaMP8‐expressing rats are well suited for both micro‐ and macro‐imaging of multiple brain regions. Because GCaMP8 expression in the parental line is controlled by the Cre–loxP system, the parental floxed GCaMP8 rat can also be used for calcium imaging of other neuronal subtypes [27, 28, 29]. For example, crossing them with rats expressing Cre under GAD or VGAT promoters would result in offspring expressing GCaMP8 in inhibitory GABAergic neurons in the neocortex [30]. An additional advantage of this approach is that the Cre–loxP system permits temporally or pharmacologically controlled expression of GCaMP, using tamoxifen‐inducible or tetracycline (Tet)‐inducible Cre‐driver lines [31]. This strategy may help avoid the developmental deficits associated with constitutive GCaMP expression, as previously reported in GCaMP3 transgenic mice [32]. In these respects, the present floxed GCaMP8 knock‐in rats represent a highly versatile tool, enabling monitoring of neuronal activity in diverse cell types and/or within specific time windows. Although Thy1 promoter‐driven GCaMP6f transgenic rats have been established previously by other groups, they do not offer such flexibility with the given constitutive expression of GCaMP6 in projection neurons [33].

Several transgenic mouse lines have been developed that express these calcium sensors in brain neurons [34, 35, 36, 37]. In this study, we compared the properties of GCaMP signals reported in previous studies with those obtained from the present Thy1–Cre–driven GCaMP8‐expressing rats, to discuss the advantages and limitations of this new rat model. Chen et al. generated a Thy1‐promoter‐driven GCaMP3 transgenic mouse line [38]. In both the present Thy1–Cre–driven GCaMP8‐expressing rats and the Thy1 promoter–driven GCaMP3 mice, the highest levels of GCaMP expression were observed in pyramidal neurons of the neocortex, hippocampus, and thalamus. In these GCaMP3 mice, in vivo two‐photon Ca^2+^ imaging revealed robust fluorescence changes ranging from 30% to 150% ΔF/F₀, which are substantially larger than those detected in the present study using transcranial macro‐imaging. Regarding the dynamic range of Ca^2+^‐dependent GCaMP fluorescence, transcranial macro‐imaging is inherently inferior, likely because of light scattering and skull autofluorescence. Dana et al. [35] generated similar transgenic mouse lines expressing GCaMP6 and performed in vivo imaging from the visual cortex [35]. They compared GCaMP expression levels in transgenic mice with those achieved via adeno‐associated virus (AAV)–mediated expression [35]. Although the expression level of GCaMP6 in the transgenic mice was lower, it was more beneficial than that obtained with the AAV vector with respect to their side effects on neuronal damage. Unfortunately, direct comparison of GCaMP fluorescence intensity between the Thy1–GCaMP6 transgenic mice and the present Thy1–Cre‐driven GCaMP8‐expressing rats is not possible. The same group also generated a mouse line expressing the red‐fluorescent calcium indicator jRGECO1a under control of the Thy1 promoter [36]. This line was used for widefield imaging of cortical activity through the intact skull, similar to the approach used in the present study. Red‐light excitation provides advantages for transcranial imaging, including improved tissue penetration and reduced hemoglobin absorption; however, the signal‐to‐noise ratio of jRGECO1a appears lower than that of conventional GCaMP indicators [36]. Consistent with these reports, we observed low but detectable hemodynamic artifacts in the present macro‐imaging experiments, necessitating image averaging to obtain clear calcium‐response maps of neuronal origin [39].

Accordingly, several limitations and technical considerations should be noted in the present study. First, the transcranial imaging approach is feasible only up to approximately 8 weeks of age in rats. Beyond this age, increased skull calcification markedly reduces light penetration [14]. As an alternative, skull thinning using a cranial drill can improve light transmittance without the need for implantation of a glass window [15]. Second, the presence of the skull causes substantial light scattering of GCaMP fluorescence, which limits spatial resolution. As a result, layer‐specific cortical responses cannot be resolved, and the recorded signals are likely to reflect averaged activity predominantly from superficial cortical layers. Third, although newer generations of calcium sensors, such as jGCaMP7/8 and XCaMPs, exhibit faster calcium kinetics that enable visualization of rapid neuronal responses in vivo [40, 41, 42], transcranial monitoring of rapid neuronal responses remains challenging in the present GCaMP8‐expressing rat line for the reasons described above. Both rat lines established and characterized in this study are freely available to researchers conducting cortical functional mapping or imaging in rats through the National BioResource Project in Japan. We hope that the present technical information will facilitate future studies on cortical functional imaging in these transgenic rats.

## Author Contributions

T.M., H. Namba, S.I., and H. Nawa analyzed the phenotypes of the recombinant rats, and M.K., K.K., N.M., T.M., R.N., and K.S. produced the parental recombinant rat lines. R.H. provided technical assistance, and H. Nawa wrote the manuscript.

## Funding

This work was supported by JSPS KAKENHI for JP 21 K18242, JP 18 K06518, and JP 22H04922 (AdAMS).

## Ethics Statement

The authors have nothing to report.

## Consent

The authors have nothing to report.

## Conflicts of Interest

H.N. receives a collaborative research grant from Otsuka Pharmaceutical Co. Ltd., and all the authors declare no conflicts of interest.

## Supporting information


**Data S1:** npr270113‐sup‐0001‐dataS1.zip.

**Data S2:** npr270113‐sup‐0002‐dataS2.zip.

**Data S3:** npr270113‐sup‐0003‐dataS3.zip.

## Data Availability

The transgenic rat lines generated in this study are freely available from the National Bioresource Project for the Rat (Japan). The original, non–contrast‐adjusted and non‐cropped raw TIFF images of the immunostaining results are provided as Data S1: #2–#10. The LabVIEW program used to operate the stimulation triggers and image acquisition systems is provided as Data S2: #11. Image datasets averaged across trials (multipage TIFF format), which form the basis of the figures presented in this study, are included as Data S3: #12. The original raw calcium imaging data (TDMS format), as well as the corresponding TIFF‐formatted images, are available in the public data repository Zenodo (https://doi.org/10.5281/zenodo.18765534 or https://doi.org/10.5281/zenodo.18765533).
