# Supplementary material for: Transcranial Calcium Macro‐Imaging From the Auditory Cortex of Thy1‐Cre‐Driven GCaMP8 Transgenic Rats
Source: Neuropsychopharmacol Rep. 2026 Apr 28;46(2):e70113. doi: 10.1002/npr2.70113 (PMC13124658; doi:10.1002/npr2.70113)
Supplement: Supplementary file 1 — Data S1: npr270113‐sup‐0001‐dataS1.zip. [file NPR2-46-e70113-s002.zip › npr270113-sup-0001-FgureS1.pdf]

Supplemental Figure

M. Tohmi et al:
Transcranial Calcium Macro-Imaging from the Auditory Cortex of Thy1-Cre-driven GCaMP8 Transgenic Rats

Figure S1: PCR identified two transgenic rat lines among the offspring born from DNA-injected eggs

Figure S1: PCR identified two transgenic rat lines among the offspring born from DNA-injected eggs

1st Screening

PCR with the primers of bX493 & X27 produce 462 bp DNA products in its genomic integration

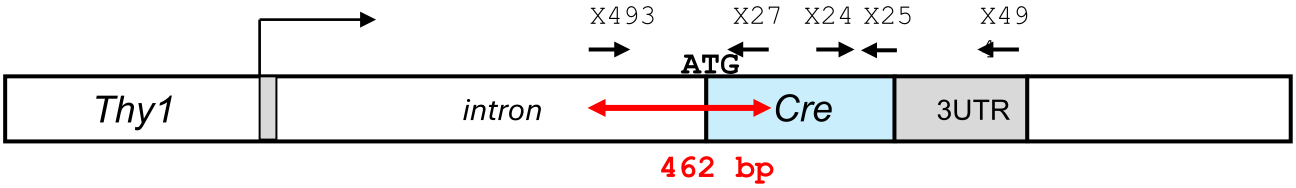

Gel electrophoresis of PCR products from rat offspring lines (#1~#33) born from

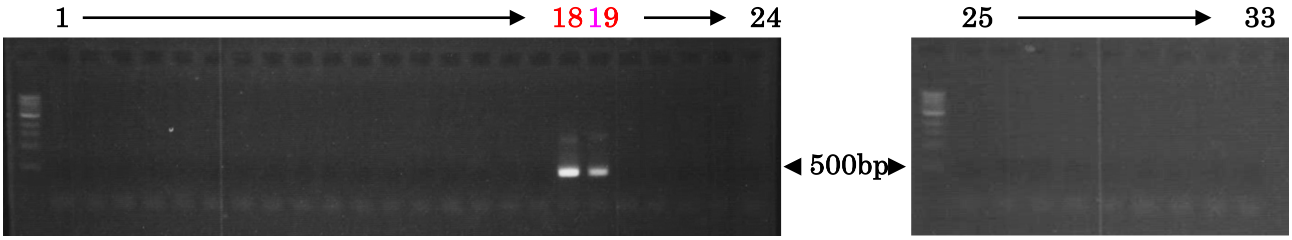

2nd Screening

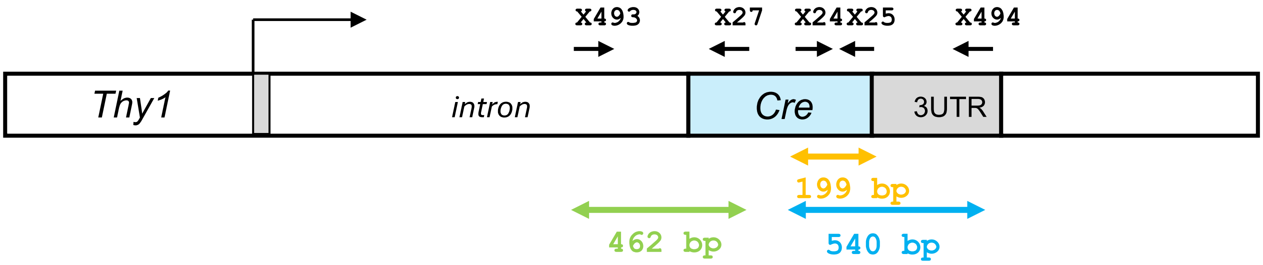

Gel electrophoresis of PCR products from rat offspring lines (#17- #20) using different primer sets

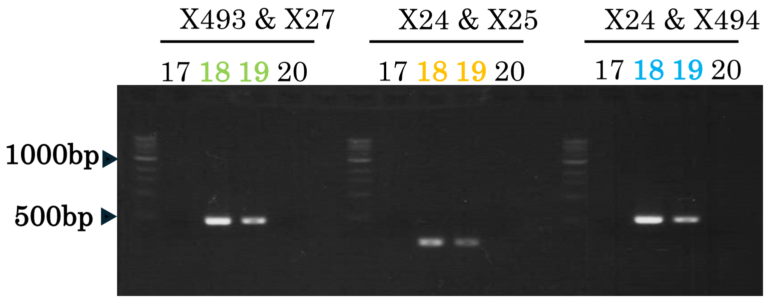

Subsequent qPCR from DNA suggested the copy number of the transgene; 5 copies in line #18 and one copy in line #19.
