# Supplementary figures and images for: Transcranial Calcium Macro‐Imaging From the Auditory Cortex of Thy1‐Cre‐Driven GCaMP8 Transgenic Rats (part 1 of 2)
Source: Neuropsychopharmacol Rep. 2026 Apr 28;46(2):e70113. doi: 10.1002/npr2.70113 (PMC13124658; doi:10.1002/npr2.70113)

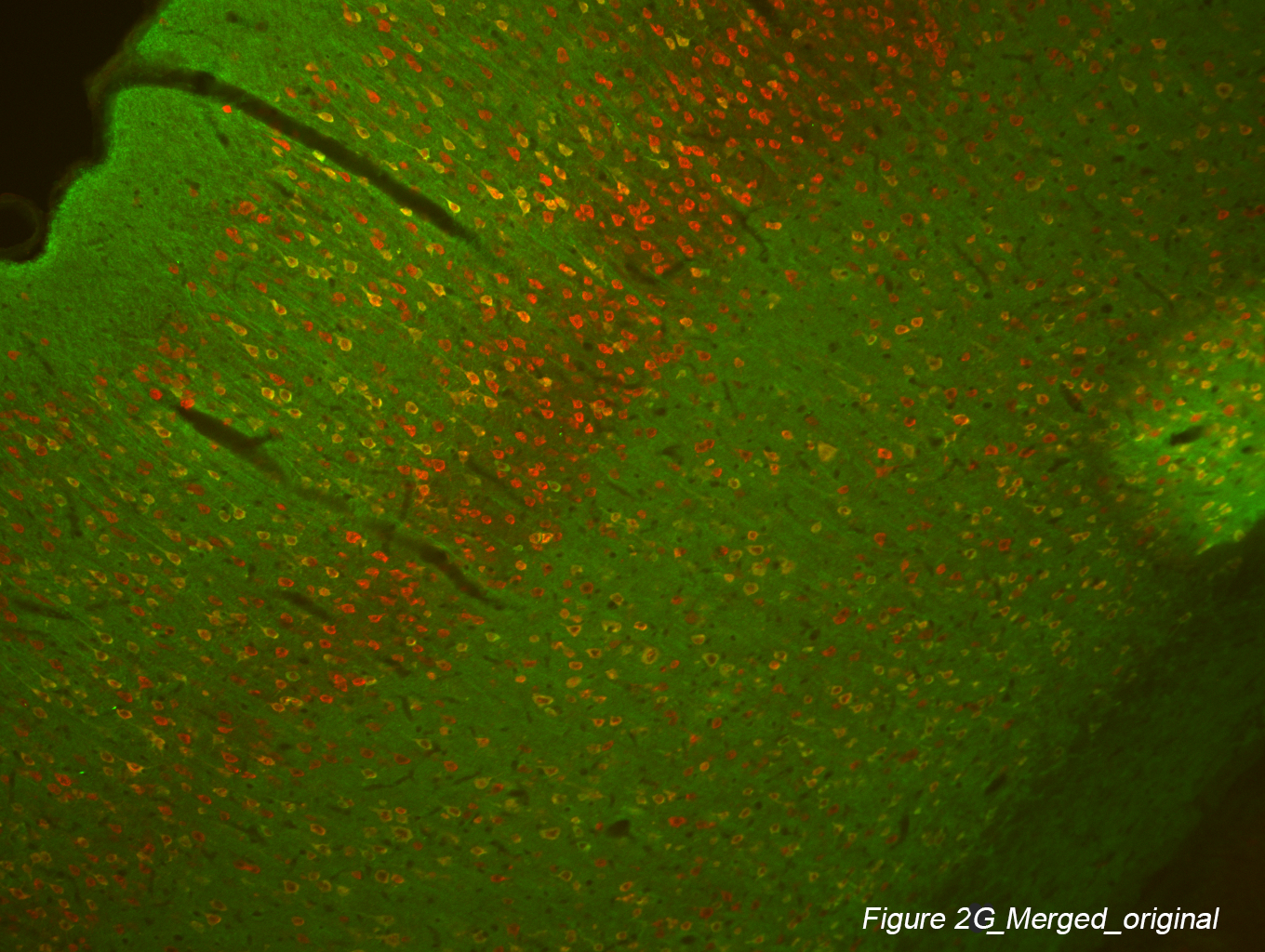

Supplement: Supplementary file 1 — Data S1: npr270113‐sup‐0001‐dataS1.zip. [file NPR2-46-e70113-s002.zip › npr270113-sup-0010-Figure 2G.TIF]

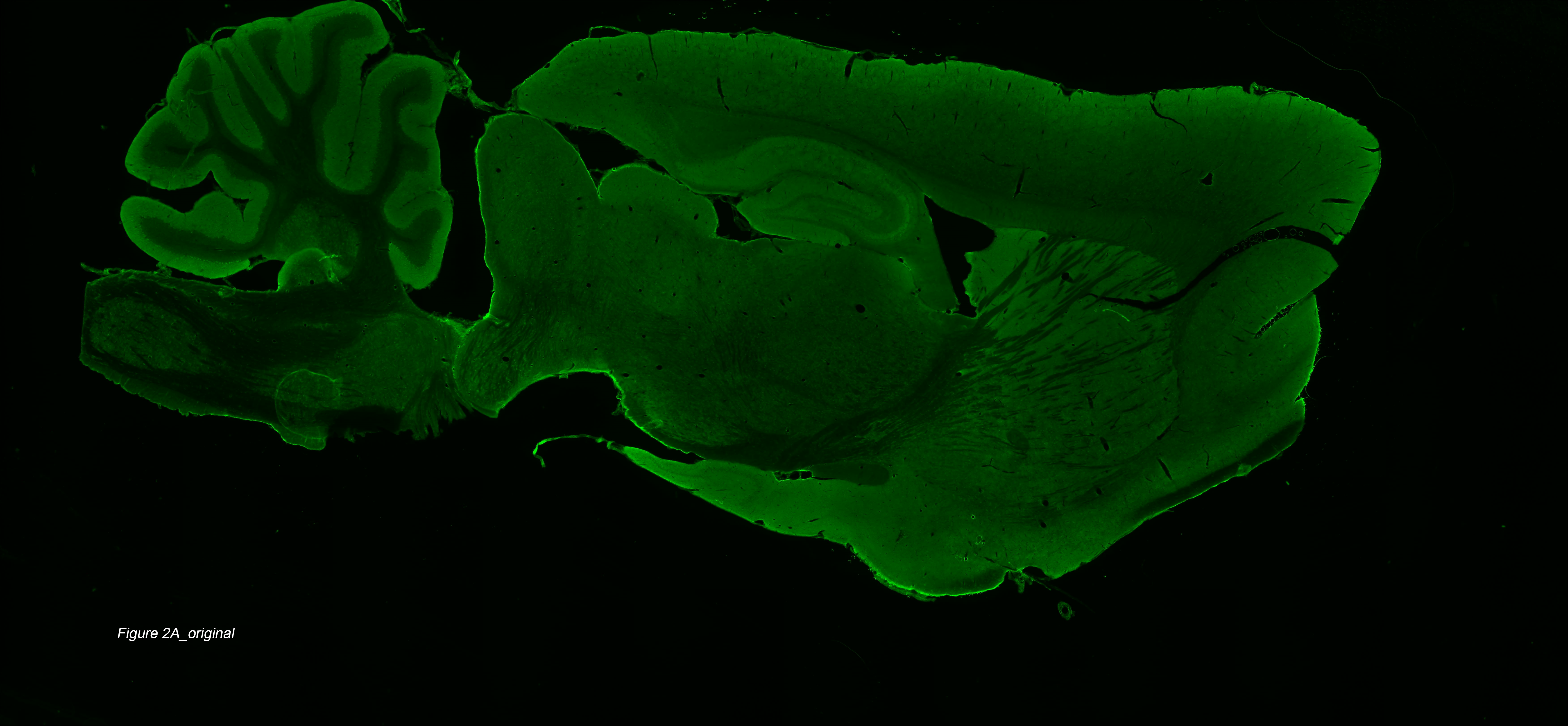

Supplement: Supplementary file 1 — Data S1: npr270113‐sup‐0001‐dataS1.zip. [file NPR2-46-e70113-s002.zip › npr270113-sup-0002-Figure 2A.tif]

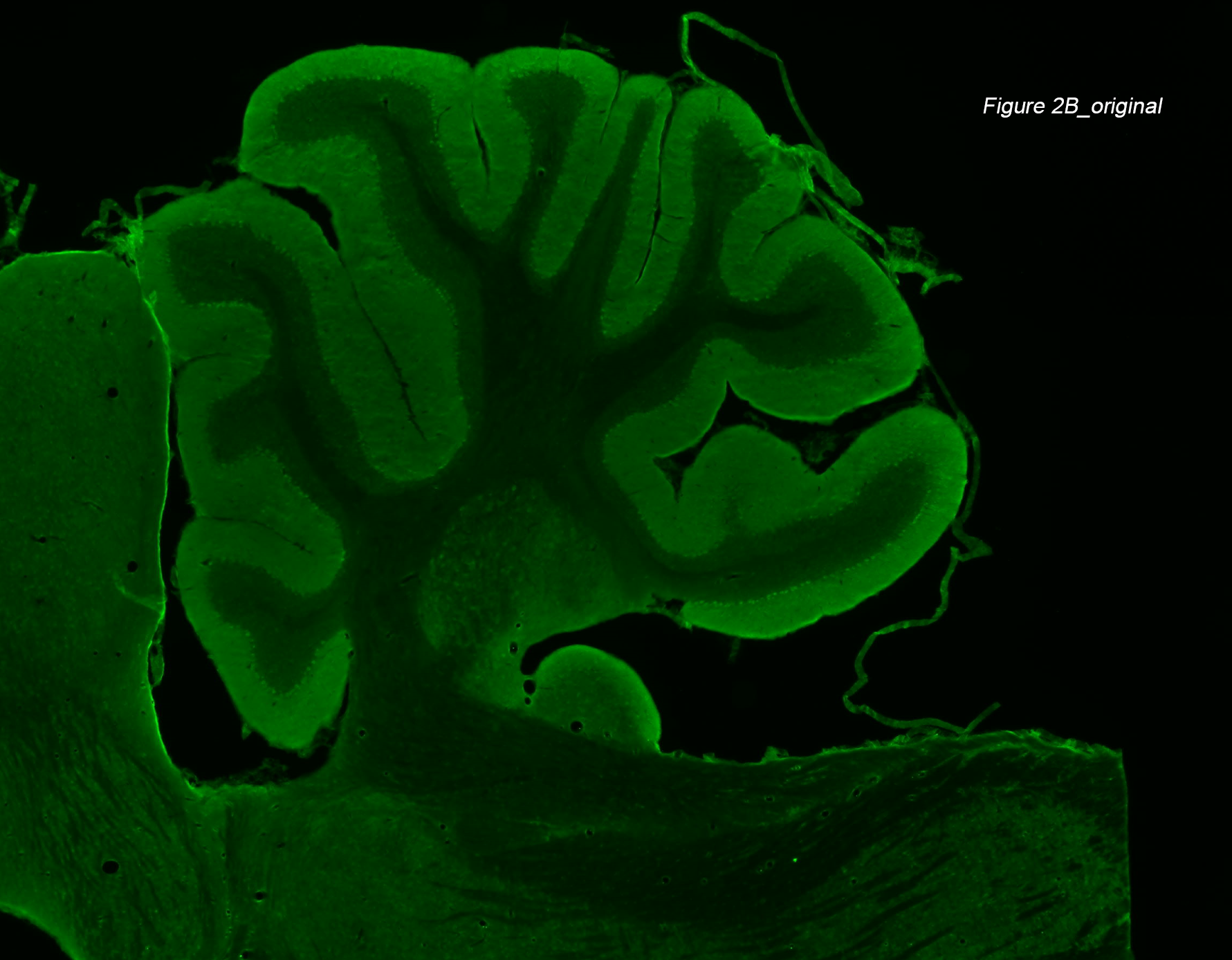

Supplement: Supplementary file 1 — Data S1: npr270113‐sup‐0001‐dataS1.zip. [file NPR2-46-e70113-s002.zip › npr270113-sup-0003-Figure 2B.tif]

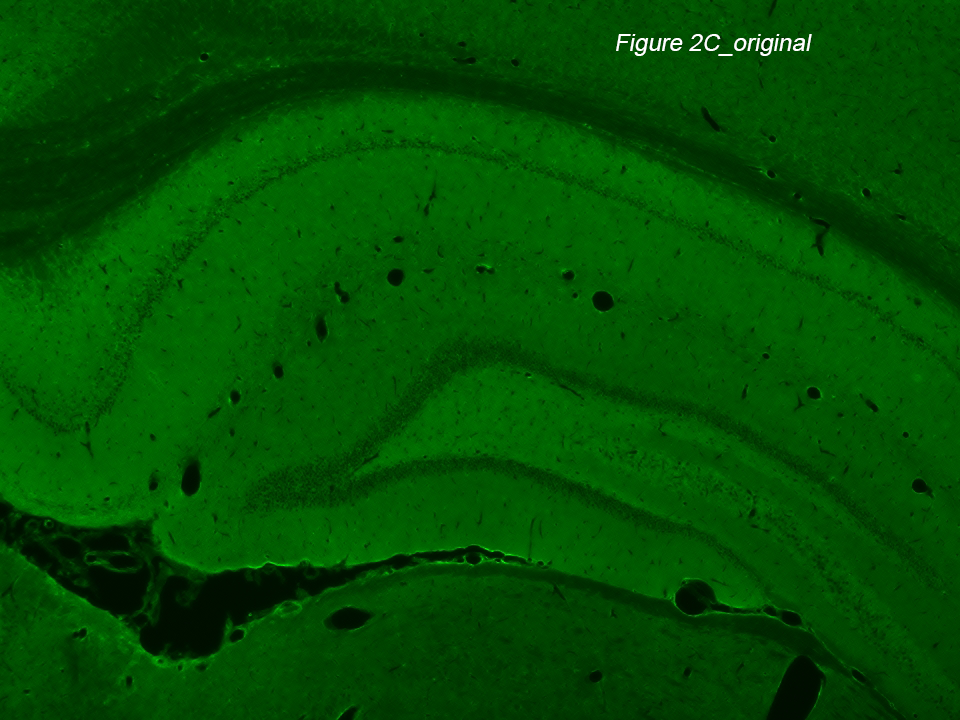

Supplement: Supplementary file 1 — Data S1: npr270113‐sup‐0001‐dataS1.zip. [file NPR2-46-e70113-s002.zip › npr270113-sup-0004-Figure 2C.tif]

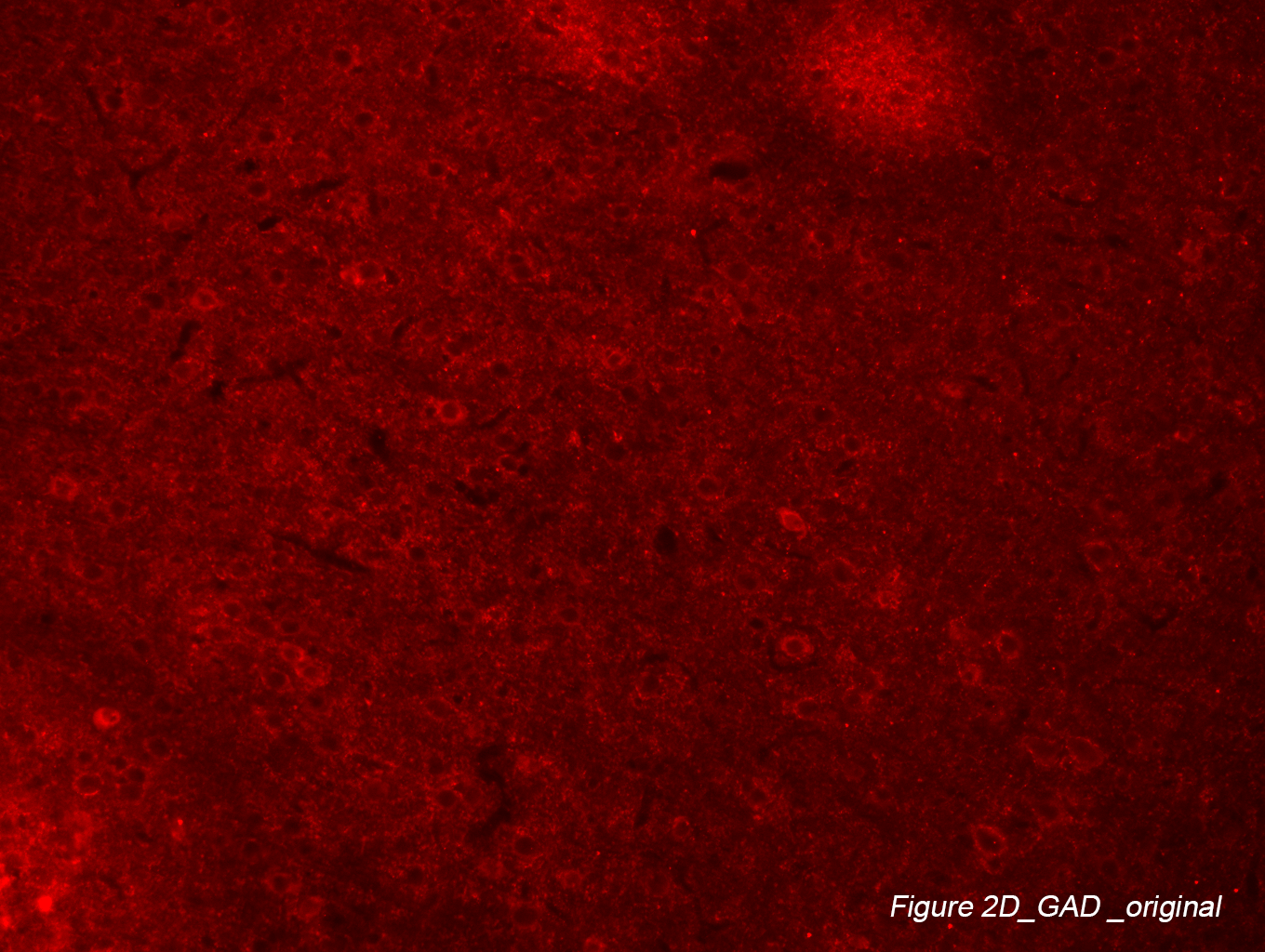

Supplement: Supplementary file 1 — Data S1: npr270113‐sup‐0001‐dataS1.zip. [file NPR2-46-e70113-s002.zip › npr270113-sup-0005-Figure 2D(a)-GAD.tif]

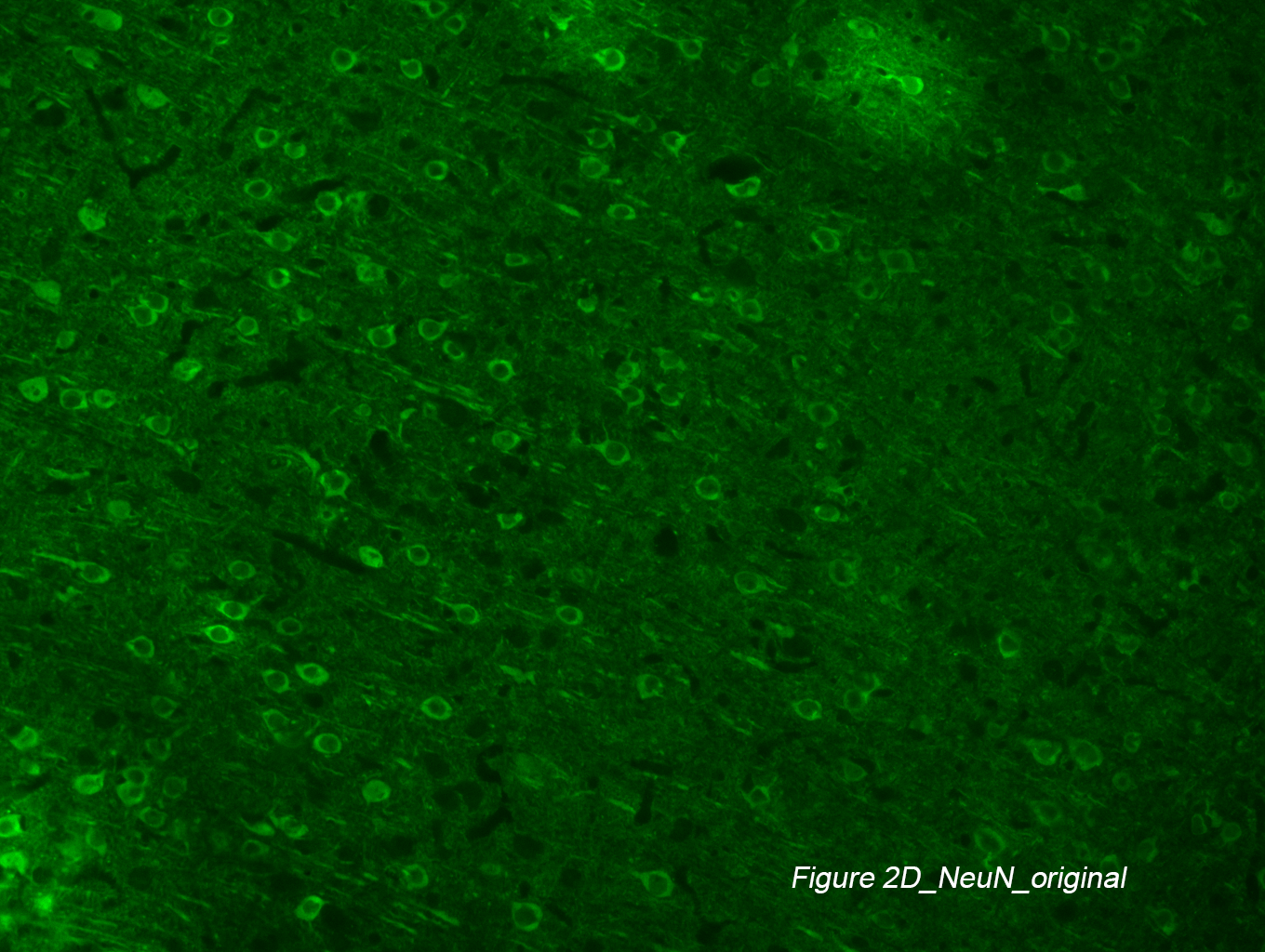

Supplement: Supplementary file 1 — Data S1: npr270113‐sup‐0001‐dataS1.zip. [file NPR2-46-e70113-s002.zip › npr270113-sup-0006-Figure 2D(b)-NeuN.TIF]

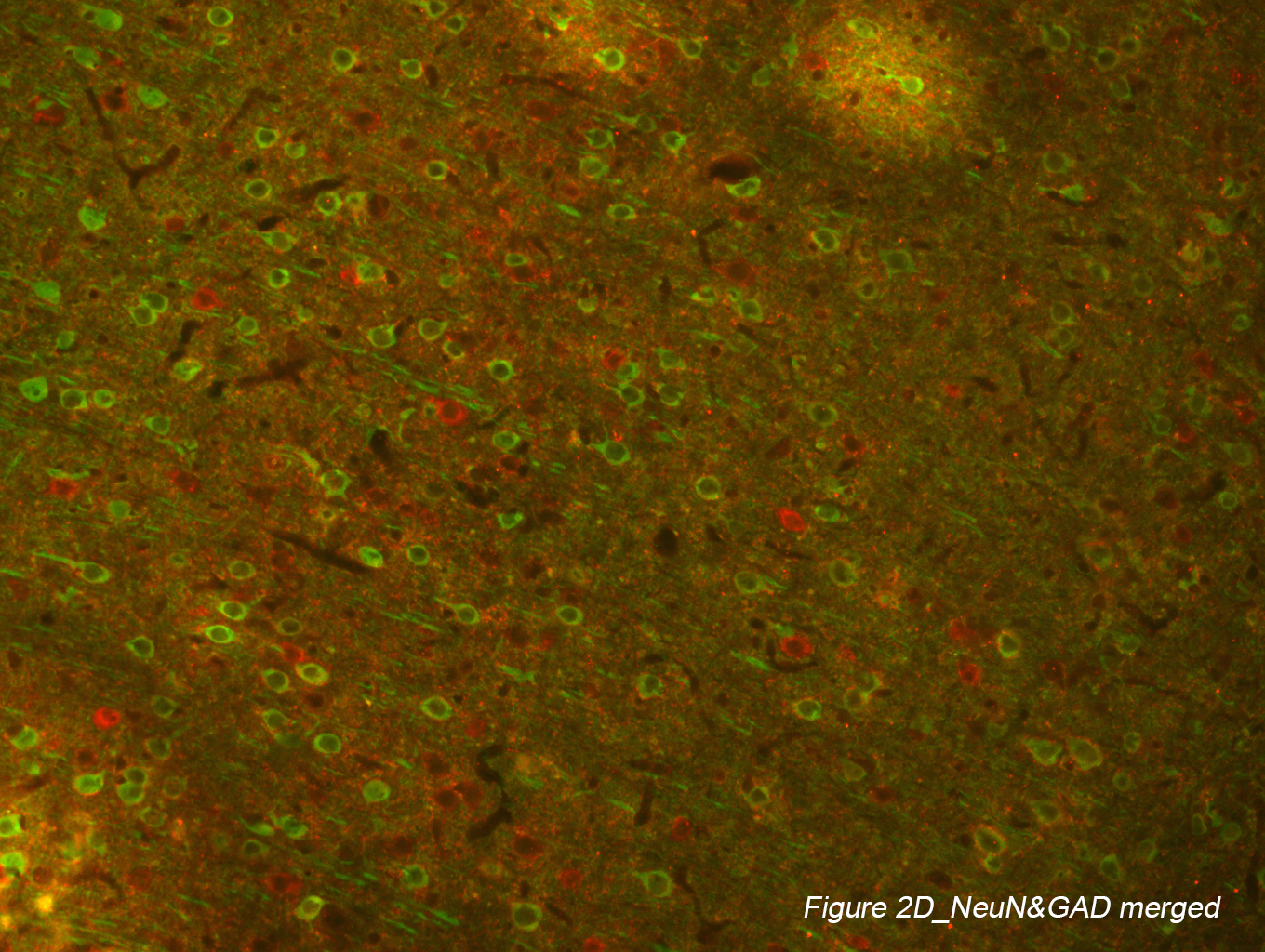

Supplement: Supplementary file 1 — Data S1: npr270113‐sup‐0001‐dataS1.zip. [file NPR2-46-e70113-s002.zip › npr270113-sup-0007-Figure 2D(c)-Merged.TIF]

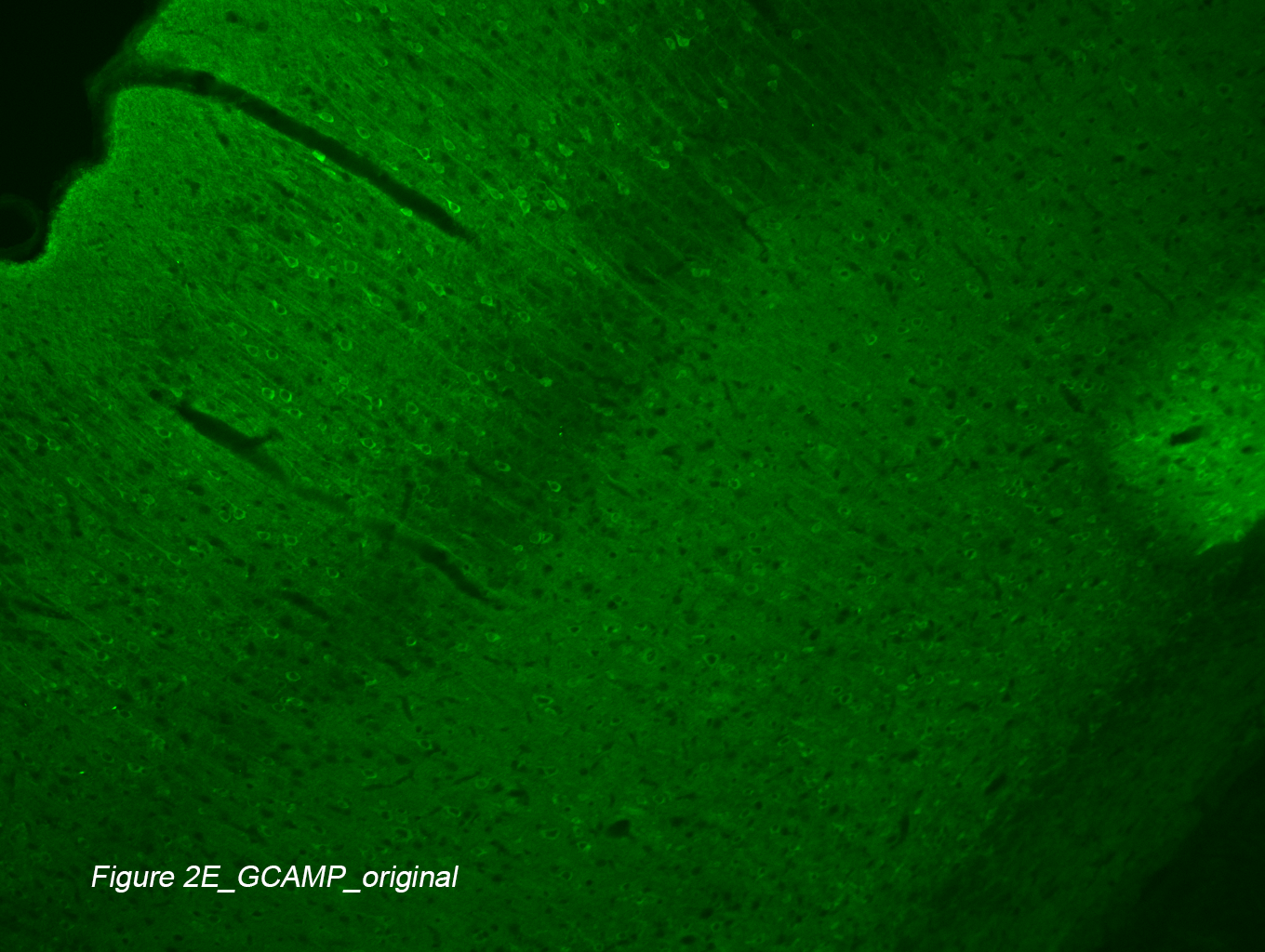

Supplement: Supplementary file 1 — Data S1: npr270113‐sup‐0001‐dataS1.zip. [file NPR2-46-e70113-s002.zip › npr270113-sup-0008-Figure 2E.TIF]

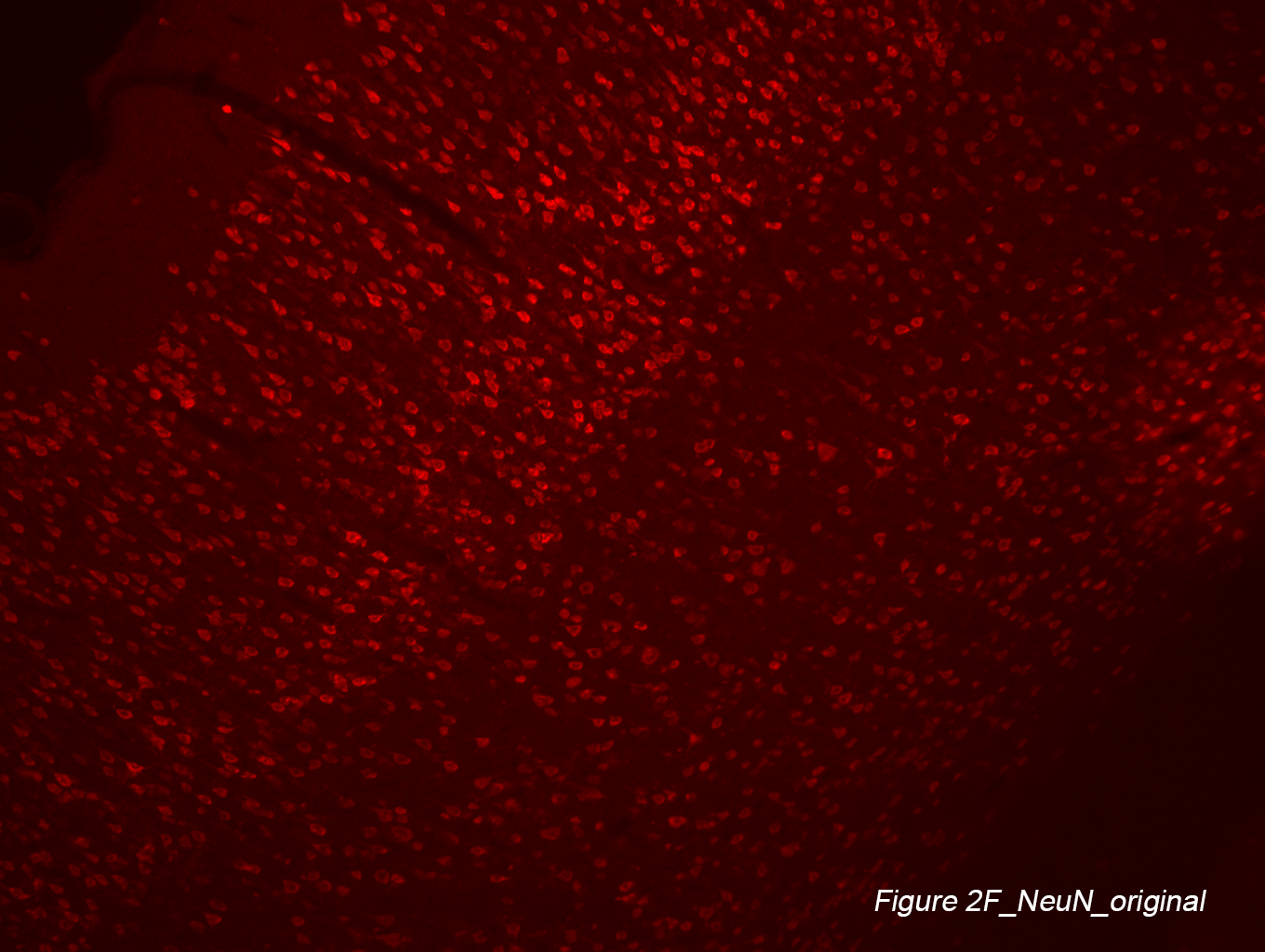

Supplement: Supplementary file 1 — Data S1: npr270113‐sup‐0001‐dataS1.zip. [file NPR2-46-e70113-s002.zip › npr270113-sup-0009-Figure 2F.TIF]

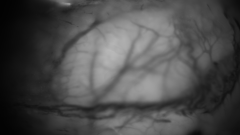

Supplement: Supplementary file 3 — Data S3: npr270113‐sup‐0003‐dataS3.zip. [file NPR2-46-e70113-s001.zip › Event Related Raw Tiff/Animal1_42db_ave.tif]

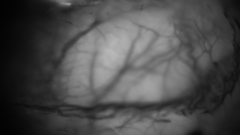

Supplement: Supplementary file 3 — Data S3: npr270113‐sup‐0003‐dataS3.zip. [file NPR2-46-e70113-s001.zip › Event Related Raw Tiff/Animal1_44db_ave.tif]

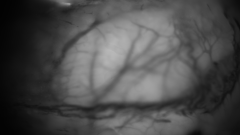

Supplement: Supplementary file 3 — Data S3: npr270113‐sup‐0003‐dataS3.zip. [file NPR2-46-e70113-s001.zip › Event Related Raw Tiff/Animal1_46db_ave.tif]

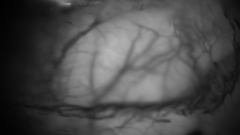

Supplement: Supplementary file 3 — Data S3: npr270113‐sup‐0003‐dataS3.zip. [file NPR2-46-e70113-s001.zip › Event Related Raw Tiff/Animal1_48db_ave.tif]

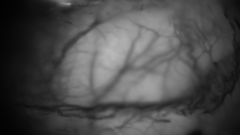

Supplement: Supplementary file 3 — Data S3: npr270113‐sup‐0003‐dataS3.zip. [file NPR2-46-e70113-s001.zip › Event Related Raw Tiff/Animal1_50db_ave.tif]

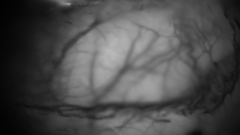

Supplement: Supplementary file 3 — Data S3: npr270113‐sup‐0003‐dataS3.zip. [file NPR2-46-e70113-s001.zip › Event Related Raw Tiff/Animal1_52db_ave.tif]

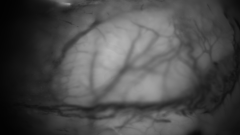

Supplement: Supplementary file 3 — Data S3: npr270113‐sup‐0003‐dataS3.zip. [file NPR2-46-e70113-s001.zip › Event Related Raw Tiff/Animal1_54db_ave.tif]

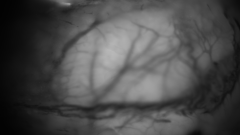

Supplement: Supplementary file 3 — Data S3: npr270113‐sup‐0003‐dataS3.zip. [file NPR2-46-e70113-s001.zip › Event Related Raw Tiff/Animal1_56db_ave.tif]

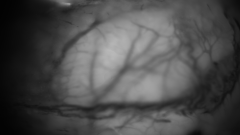

Supplement: Supplementary file 3 — Data S3: npr270113‐sup‐0003‐dataS3.zip. [file NPR2-46-e70113-s001.zip › Event Related Raw Tiff/Animal1_58db_ave.tif]

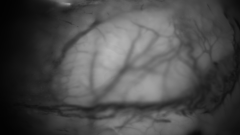

Supplement: Supplementary file 3 — Data S3: npr270113‐sup‐0003‐dataS3.zip. [file NPR2-46-e70113-s001.zip › Event Related Raw Tiff/Animal1_60db_ave.tif]

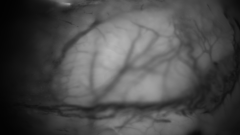

Supplement: Supplementary file 3 — Data S3: npr270113‐sup‐0003‐dataS3.zip. [file NPR2-46-e70113-s001.zip › Event Related Raw Tiff/Animal1_62db_ave.tif]

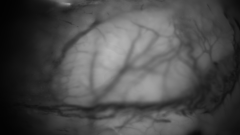

Supplement: Supplementary file 3 — Data S3: npr270113‐sup‐0003‐dataS3.zip. [file NPR2-46-e70113-s001.zip › Event Related Raw Tiff/Animal1_64db_ave.tif]

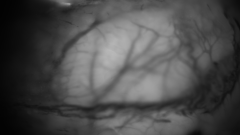

Supplement: Supplementary file 3 — Data S3: npr270113‐sup‐0003‐dataS3.zip. [file NPR2-46-e70113-s001.zip › Event Related Raw Tiff/Animal1_66db_ave.tif]

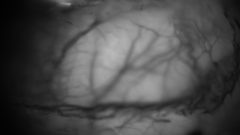

Supplement: Supplementary file 3 — Data S3: npr270113‐sup‐0003‐dataS3.zip. [file NPR2-46-e70113-s001.zip › Event Related Raw Tiff/Animal1_68db_ave.tif]

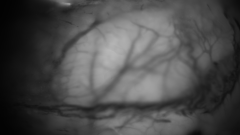

Supplement: Supplementary file 3 — Data S3: npr270113‐sup‐0003‐dataS3.zip. [file NPR2-46-e70113-s001.zip › Event Related Raw Tiff/Animal1_70db_ave.tif]

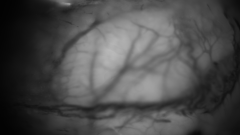

Supplement: Supplementary file 3 — Data S3: npr270113‐sup‐0003‐dataS3.zip. [file NPR2-46-e70113-s001.zip › Event Related Raw Tiff/Animal1_72db_ave.tif]

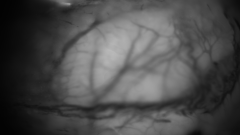

Supplement: Supplementary file 3 — Data S3: npr270113‐sup‐0003‐dataS3.zip. [file NPR2-46-e70113-s001.zip › Event Related Raw Tiff/Animal1_74db_ave.tif]

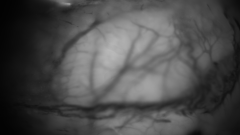

Supplement: Supplementary file 3 — Data S3: npr270113‐sup‐0003‐dataS3.zip. [file NPR2-46-e70113-s001.zip › Event Related Raw Tiff/Animal1_76db_ave.tif]

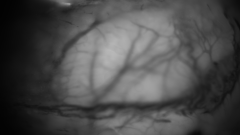

Supplement: Supplementary file 3 — Data S3: npr270113‐sup‐0003‐dataS3.zip. [file NPR2-46-e70113-s001.zip › Event Related Raw Tiff/Animal1_78db_ave.tif]

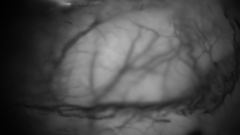

Supplement: Supplementary file 3 — Data S3: npr270113‐sup‐0003‐dataS3.zip. [file NPR2-46-e70113-s001.zip › Event Related Raw Tiff/Animal1_80db_ave.tif]

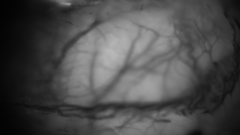

Supplement: Supplementary file 3 — Data S3: npr270113‐sup‐0003‐dataS3.zip. [file NPR2-46-e70113-s001.zip › Event Related Raw Tiff/Animal1_82db_ave.tif]

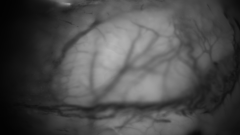

Supplement: Supplementary file 3 — Data S3: npr270113‐sup‐0003‐dataS3.zip. [file NPR2-46-e70113-s001.zip › Event Related Raw Tiff/Animal1_84db_ave.tif]

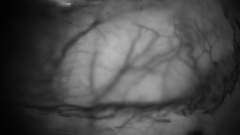

Supplement: Supplementary file 3 — Data S3: npr270113‐sup‐0003‐dataS3.zip. [file NPR2-46-e70113-s001.zip › Event Related Raw Tiff/Animal1_86db_ave.tif]

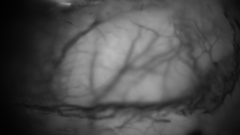

Supplement: Supplementary file 3 — Data S3: npr270113‐sup‐0003‐dataS3.zip. [file NPR2-46-e70113-s001.zip › Event Related Raw Tiff/Animal1_88db_ave.tif]

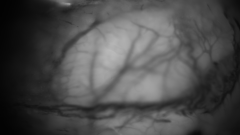

Supplement: Supplementary file 3 — Data S3: npr270113‐sup‐0003‐dataS3.zip. [file NPR2-46-e70113-s001.zip › Event Related Raw Tiff/Animal1_90db_ave.tif]

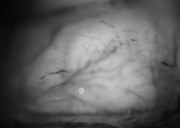

Supplement: Supplementary file 3 — Data S3: npr270113‐sup‐0003‐dataS3.zip. [file NPR2-46-e70113-s001.zip › Event Related Raw Tiff/Animal2_42db_ave.tif]

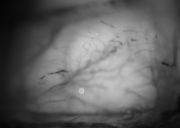

Supplement: Supplementary file 3 — Data S3: npr270113‐sup‐0003‐dataS3.zip. [file NPR2-46-e70113-s001.zip › Event Related Raw Tiff/Animal2_44db_ave.tif]

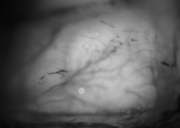

Supplement: Supplementary file 3 — Data S3: npr270113‐sup‐0003‐dataS3.zip. [file NPR2-46-e70113-s001.zip › Event Related Raw Tiff/Animal2_46db_ave.tif]

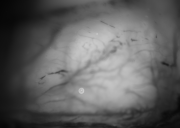

Supplement: Supplementary file 3 — Data S3: npr270113‐sup‐0003‐dataS3.zip. [file NPR2-46-e70113-s001.zip › Event Related Raw Tiff/Animal2_48db_ave.tif]

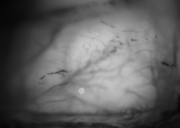

Supplement: Supplementary file 3 — Data S3: npr270113‐sup‐0003‐dataS3.zip. [file NPR2-46-e70113-s001.zip › Event Related Raw Tiff/Animal2_50db_ave.tif]

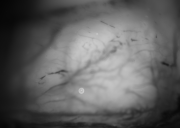

Supplement: Supplementary file 3 — Data S3: npr270113‐sup‐0003‐dataS3.zip. [file NPR2-46-e70113-s001.zip › Event Related Raw Tiff/Animal2_52db_ave.tif]

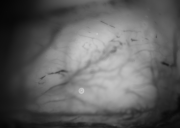

Supplement: Supplementary file 3 — Data S3: npr270113‐sup‐0003‐dataS3.zip. [file NPR2-46-e70113-s001.zip › Event Related Raw Tiff/Animal2_54db_ave.tif]

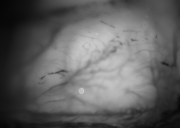

Supplement: Supplementary file 3 — Data S3: npr270113‐sup‐0003‐dataS3.zip. [file NPR2-46-e70113-s001.zip › Event Related Raw Tiff/Animal2_56db_ave.tif]

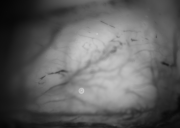

Supplement: Supplementary file 3 — Data S3: npr270113‐sup‐0003‐dataS3.zip. [file NPR2-46-e70113-s001.zip › Event Related Raw Tiff/Animal2_58db_ave.tif]

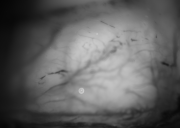

Supplement: Supplementary file 3 — Data S3: npr270113‐sup‐0003‐dataS3.zip. [file NPR2-46-e70113-s001.zip › Event Related Raw Tiff/Animal2_60db_ave.tif]

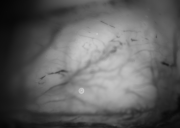

Supplement: Supplementary file 3 — Data S3: npr270113‐sup‐0003‐dataS3.zip. [file NPR2-46-e70113-s001.zip › Event Related Raw Tiff/Animal2_62db_ave.tif]

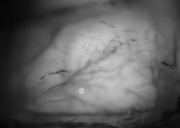

Supplement: Supplementary file 3 — Data S3: npr270113‐sup‐0003‐dataS3.zip. [file NPR2-46-e70113-s001.zip › Event Related Raw Tiff/Animal2_64db_ave.tif]

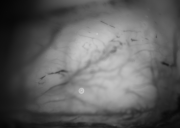

Supplement: Supplementary file 3 — Data S3: npr270113‐sup‐0003‐dataS3.zip. [file NPR2-46-e70113-s001.zip › Event Related Raw Tiff/Animal2_66db_ave.tif]

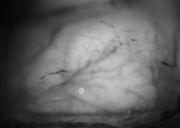

Supplement: Supplementary file 3 — Data S3: npr270113‐sup‐0003‐dataS3.zip. [file NPR2-46-e70113-s001.zip › Event Related Raw Tiff/Animal2_68db_ave.tif]

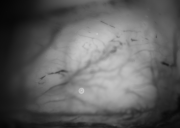

Supplement: Supplementary file 3 — Data S3: npr270113‐sup‐0003‐dataS3.zip. [file NPR2-46-e70113-s001.zip › Event Related Raw Tiff/Animal2_70db_ave.tif]

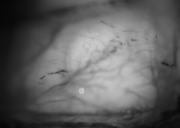

Supplement: Supplementary file 3 — Data S3: npr270113‐sup‐0003‐dataS3.zip. [file NPR2-46-e70113-s001.zip › Event Related Raw Tiff/Animal2_72db_ave.tif]

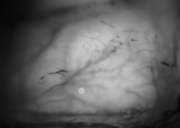

Supplement: Supplementary file 3 — Data S3: npr270113‐sup‐0003‐dataS3.zip. [file NPR2-46-e70113-s001.zip › Event Related Raw Tiff/Animal2_74db_ave.tif]

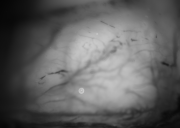

Supplement: Supplementary file 3 — Data S3: npr270113‐sup‐0003‐dataS3.zip. [file NPR2-46-e70113-s001.zip › Event Related Raw Tiff/Animal2_76db_ave.tif]

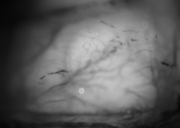

Supplement: Supplementary file 3 — Data S3: npr270113‐sup‐0003‐dataS3.zip. [file NPR2-46-e70113-s001.zip › Event Related Raw Tiff/Animal2_78db_ave.tif]

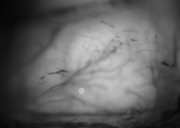

Supplement: Supplementary file 3 — Data S3: npr270113‐sup‐0003‐dataS3.zip. [file NPR2-46-e70113-s001.zip › Event Related Raw Tiff/Animal2_80db_ave.tif]

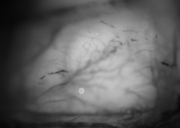

Supplement: Supplementary file 3 — Data S3: npr270113‐sup‐0003‐dataS3.zip. [file NPR2-46-e70113-s001.zip › Event Related Raw Tiff/Animal2_82db_ave.tif]

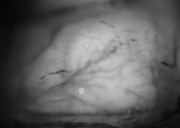

Supplement: Supplementary file 3 — Data S3: npr270113‐sup‐0003‐dataS3.zip. [file NPR2-46-e70113-s001.zip › Event Related Raw Tiff/Animal2_84db_ave.tif]

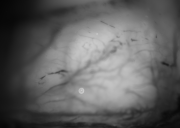

Supplement: Supplementary file 3 — Data S3: npr270113‐sup‐0003‐dataS3.zip. [file NPR2-46-e70113-s001.zip › Event Related Raw Tiff/Animal2_86db_ave.tif]

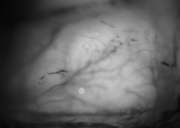

Supplement: Supplementary file 3 — Data S3: npr270113‐sup‐0003‐dataS3.zip. [file NPR2-46-e70113-s001.zip › Event Related Raw Tiff/Animal2_88db_ave.tif]

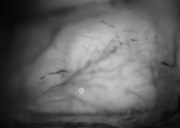

Supplement: Supplementary file 3 — Data S3: npr270113‐sup‐0003‐dataS3.zip. [file NPR2-46-e70113-s001.zip › Event Related Raw Tiff/Animal2_90db_ave.tif]

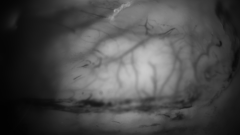

Supplement: Supplementary file 3 — Data S3: npr270113‐sup‐0003‐dataS3.zip. [file NPR2-46-e70113-s001.zip › Event Related Raw Tiff/Animal3_42db_ave.tif]

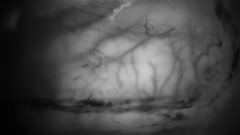

Supplement: Supplementary file 3 — Data S3: npr270113‐sup‐0003‐dataS3.zip. [file NPR2-46-e70113-s001.zip › Event Related Raw Tiff/Animal3_44db_ave.tif]

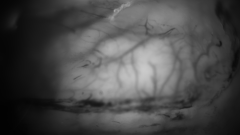

Supplement: Supplementary file 3 — Data S3: npr270113‐sup‐0003‐dataS3.zip. [file NPR2-46-e70113-s001.zip › Event Related Raw Tiff/Animal3_46db_ave.tif]

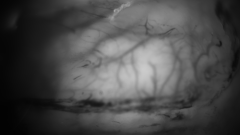

Supplement: Supplementary file 3 — Data S3: npr270113‐sup‐0003‐dataS3.zip. [file NPR2-46-e70113-s001.zip › Event Related Raw Tiff/Animal3_48db_ave.tif]

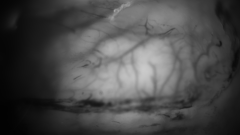

Supplement: Supplementary file 3 — Data S3: npr270113‐sup‐0003‐dataS3.zip. [file NPR2-46-e70113-s001.zip › Event Related Raw Tiff/Animal3_50db_ave.tif]

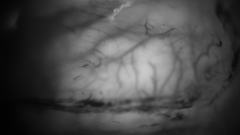

Supplement: Supplementary file 3 — Data S3: npr270113‐sup‐0003‐dataS3.zip. [file NPR2-46-e70113-s001.zip › Event Related Raw Tiff/Animal3_52db_ave.tif]

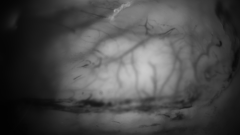

Supplement: Supplementary file 3 — Data S3: npr270113‐sup‐0003‐dataS3.zip. [file NPR2-46-e70113-s001.zip › Event Related Raw Tiff/Animal3_54db_ave.tif]

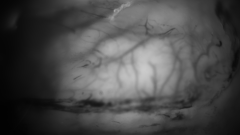

Supplement: Supplementary file 3 — Data S3: npr270113‐sup‐0003‐dataS3.zip. [file NPR2-46-e70113-s001.zip › Event Related Raw Tiff/Animal3_56db_ave.tif]

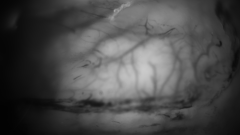

Supplement: Supplementary file 3 — Data S3: npr270113‐sup‐0003‐dataS3.zip. [file NPR2-46-e70113-s001.zip › Event Related Raw Tiff/Animal3_58db_ave.tif]

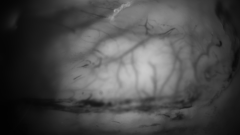

Supplement: Supplementary file 3 — Data S3: npr270113‐sup‐0003‐dataS3.zip. [file NPR2-46-e70113-s001.zip › Event Related Raw Tiff/Animal3_60db_ave.tif]

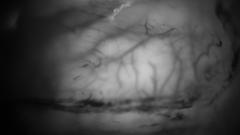

Supplement: Supplementary file 3 — Data S3: npr270113‐sup‐0003‐dataS3.zip. [file NPR2-46-e70113-s001.zip › Event Related Raw Tiff/Animal3_62db_ave.tif]

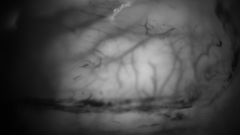

Supplement: Supplementary file 3 — Data S3: npr270113‐sup‐0003‐dataS3.zip. [file NPR2-46-e70113-s001.zip › Event Related Raw Tiff/Animal3_64db_ave.tif]

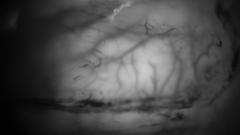

Supplement: Supplementary file 3 — Data S3: npr270113‐sup‐0003‐dataS3.zip. [file NPR2-46-e70113-s001.zip › Event Related Raw Tiff/Animal3_66db_ave.tif]

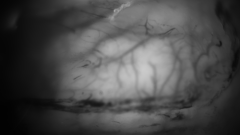

Supplement: Supplementary file 3 — Data S3: npr270113‐sup‐0003‐dataS3.zip. [file NPR2-46-e70113-s001.zip › Event Related Raw Tiff/Animal3_68db_ave.tif]

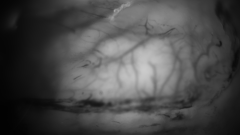

Supplement: Supplementary file 3 — Data S3: npr270113‐sup‐0003‐dataS3.zip. [file NPR2-46-e70113-s001.zip › Event Related Raw Tiff/Animal3_70db_ave.tif]

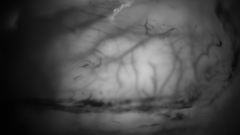

Supplement: Supplementary file 3 — Data S3: npr270113‐sup‐0003‐dataS3.zip. [file NPR2-46-e70113-s001.zip › Event Related Raw Tiff/Animal3_72db_ave.tif]

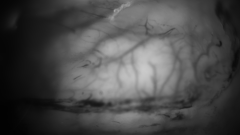

Supplement: Supplementary file 3 — Data S3: npr270113‐sup‐0003‐dataS3.zip. [file NPR2-46-e70113-s001.zip › Event Related Raw Tiff/Animal3_74db_ave.tif]

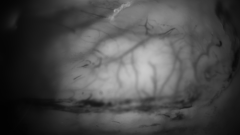

Supplement: Supplementary file 3 — Data S3: npr270113‐sup‐0003‐dataS3.zip. [file NPR2-46-e70113-s001.zip › Event Related Raw Tiff/Animal3_76db_ave.tif]

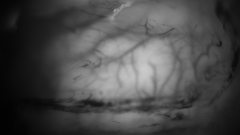

Supplement: Supplementary file 3 — Data S3: npr270113‐sup‐0003‐dataS3.zip. [file NPR2-46-e70113-s001.zip › Event Related Raw Tiff/Animal3_78db_ave.tif]

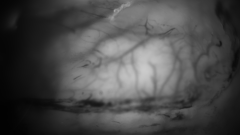

Supplement: Supplementary file 3 — Data S3: npr270113‐sup‐0003‐dataS3.zip. [file NPR2-46-e70113-s001.zip › Event Related Raw Tiff/Animal3_80db_ave.tif]

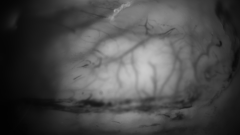

Supplement: Supplementary file 3 — Data S3: npr270113‐sup‐0003‐dataS3.zip. [file NPR2-46-e70113-s001.zip › Event Related Raw Tiff/Animal3_82db_ave.tif]

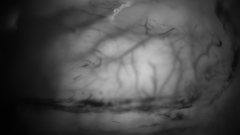

Supplement: Supplementary file 3 — Data S3: npr270113‐sup‐0003‐dataS3.zip. [file NPR2-46-e70113-s001.zip › Event Related Raw Tiff/Animal3_84db_ave.tif]

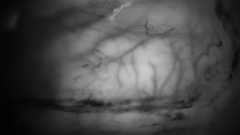

Supplement: Supplementary file 3 — Data S3: npr270113‐sup‐0003‐dataS3.zip. [file NPR2-46-e70113-s001.zip › Event Related Raw Tiff/Animal3_86db_ave.tif]

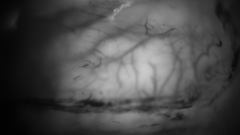

Supplement: Supplementary file 3 — Data S3: npr270113‐sup‐0003‐dataS3.zip. [file NPR2-46-e70113-s001.zip › Event Related Raw Tiff/Animal3_88db_ave.tif]

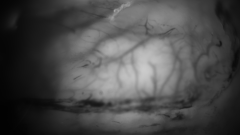

Supplement: Supplementary file 3 — Data S3: npr270113‐sup‐0003‐dataS3.zip. [file NPR2-46-e70113-s001.zip › Event Related Raw Tiff/Animal3_90db_ave.tif]

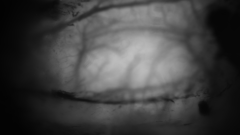

Supplement: Supplementary file 3 — Data S3: npr270113‐sup‐0003‐dataS3.zip. [file NPR2-46-e70113-s001.zip › Event Related Raw Tiff/Animal4_42db_ave.tif]

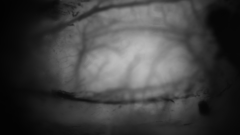

Supplement: Supplementary file 3 — Data S3: npr270113‐sup‐0003‐dataS3.zip. [file NPR2-46-e70113-s001.zip › Event Related Raw Tiff/Animal4_44db_ave.tif]

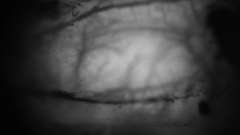

Supplement: Supplementary file 3 — Data S3: npr270113‐sup‐0003‐dataS3.zip. [file NPR2-46-e70113-s001.zip › Event Related Raw Tiff/Animal4_46db_ave.tif]

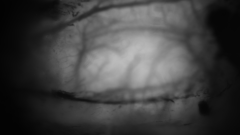

Supplement: Supplementary file 3 — Data S3: npr270113‐sup‐0003‐dataS3.zip. [file NPR2-46-e70113-s001.zip › Event Related Raw Tiff/Animal4_48db_ave.tif]

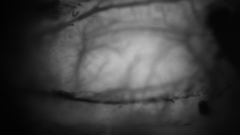

Supplement: Supplementary file 3 — Data S3: npr270113‐sup‐0003‐dataS3.zip. [file NPR2-46-e70113-s001.zip › Event Related Raw Tiff/Animal4_50db_ave.tif]

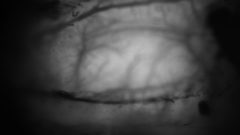

Supplement: Supplementary file 3 — Data S3: npr270113‐sup‐0003‐dataS3.zip. [file NPR2-46-e70113-s001.zip › Event Related Raw Tiff/Animal4_52db_ave.tif]

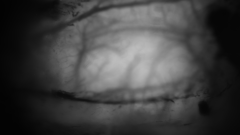

Supplement: Supplementary file 3 — Data S3: npr270113‐sup‐0003‐dataS3.zip. [file NPR2-46-e70113-s001.zip › Event Related Raw Tiff/Animal4_54db_ave.tif]

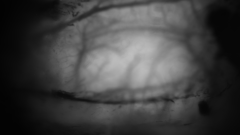

Supplement: Supplementary file 3 — Data S3: npr270113‐sup‐0003‐dataS3.zip. [file NPR2-46-e70113-s001.zip › Event Related Raw Tiff/Animal4_56db_ave.tif]

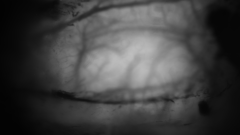

Supplement: Supplementary file 3 — Data S3: npr270113‐sup‐0003‐dataS3.zip. [file NPR2-46-e70113-s001.zip › Event Related Raw Tiff/Animal4_58db_ave.tif]

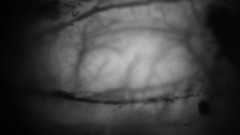

Supplement: Supplementary file 3 — Data S3: npr270113‐sup‐0003‐dataS3.zip. [file NPR2-46-e70113-s001.zip › Event Related Raw Tiff/Animal4_60db_ave.tif]

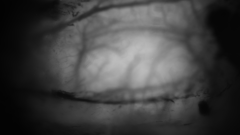

Supplement: Supplementary file 3 — Data S3: npr270113‐sup‐0003‐dataS3.zip. [file NPR2-46-e70113-s001.zip › Event Related Raw Tiff/Animal4_62db_ave.tif]

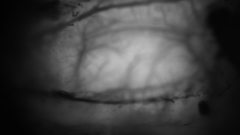

Supplement: Supplementary file 3 — Data S3: npr270113‐sup‐0003‐dataS3.zip. [file NPR2-46-e70113-s001.zip › Event Related Raw Tiff/Animal4_64db_ave.tif]

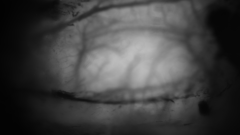

Supplement: Supplementary file 3 — Data S3: npr270113‐sup‐0003‐dataS3.zip. [file NPR2-46-e70113-s001.zip › Event Related Raw Tiff/Animal4_66db_ave.tif]

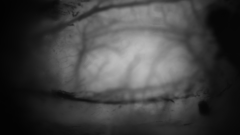

Supplement: Supplementary file 3 — Data S3: npr270113‐sup‐0003‐dataS3.zip. [file NPR2-46-e70113-s001.zip › Event Related Raw Tiff/Animal4_68db_ave.tif]

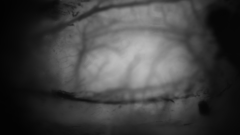

Supplement: Supplementary file 3 — Data S3: npr270113‐sup‐0003‐dataS3.zip. [file NPR2-46-e70113-s001.zip › Event Related Raw Tiff/Animal4_70db_ave.tif]

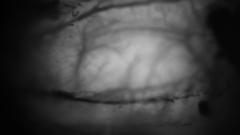

Supplement: Supplementary file 3 — Data S3: npr270113‐sup‐0003‐dataS3.zip. [file NPR2-46-e70113-s001.zip › Event Related Raw Tiff/Animal4_72db_ave.tif]
