# Supplementary figures and images for: Transcranial Calcium Macro‐Imaging From the Auditory Cortex of Thy1‐Cre‐Driven GCaMP8 Transgenic Rats (part 2 of 2)
Source: Neuropsychopharmacol Rep. 2026 Apr 28;46(2):e70113. doi: 10.1002/npr2.70113 (PMC13124658; doi:10.1002/npr2.70113)

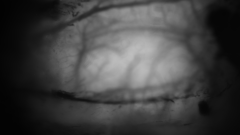

Supplement: Supplementary file 3 — Data S3: npr270113‐sup‐0003‐dataS3.zip. [file NPR2-46-e70113-s001.zip › Event Related Raw Tiff/Animal4_74db_ave.tif]

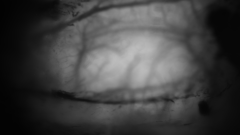

Supplement: Supplementary file 3 — Data S3: npr270113‐sup‐0003‐dataS3.zip. [file NPR2-46-e70113-s001.zip › Event Related Raw Tiff/Animal4_76db_ave.tif]

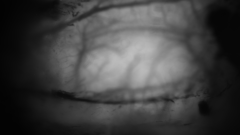

Supplement: Supplementary file 3 — Data S3: npr270113‐sup‐0003‐dataS3.zip. [file NPR2-46-e70113-s001.zip › Event Related Raw Tiff/Animal4_78db_ave.tif]

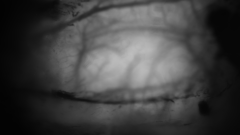

Supplement: Supplementary file 3 — Data S3: npr270113‐sup‐0003‐dataS3.zip. [file NPR2-46-e70113-s001.zip › Event Related Raw Tiff/Animal4_80db_ave.tif]

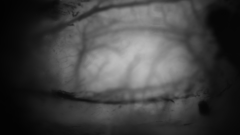

Supplement: Supplementary file 3 — Data S3: npr270113‐sup‐0003‐dataS3.zip. [file NPR2-46-e70113-s001.zip › Event Related Raw Tiff/Animal4_82db_ave.tif]

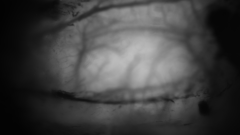

Supplement: Supplementary file 3 — Data S3: npr270113‐sup‐0003‐dataS3.zip. [file NPR2-46-e70113-s001.zip › Event Related Raw Tiff/Animal4_84db_ave.tif]

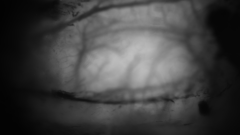

Supplement: Supplementary file 3 — Data S3: npr270113‐sup‐0003‐dataS3.zip. [file NPR2-46-e70113-s001.zip › Event Related Raw Tiff/Animal4_86db_ave.tif]

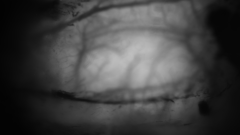

Supplement: Supplementary file 3 — Data S3: npr270113‐sup‐0003‐dataS3.zip. [file NPR2-46-e70113-s001.zip › Event Related Raw Tiff/Animal4_88db_ave.tif]

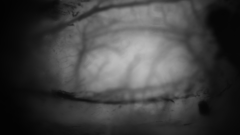

Supplement: Supplementary file 3 — Data S3: npr270113‐sup‐0003‐dataS3.zip. [file NPR2-46-e70113-s001.zip › Event Related Raw Tiff/Animal4_90db_ave.tif]

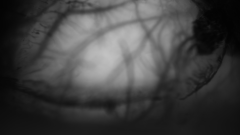

Supplement: Supplementary file 3 — Data S3: npr270113‐sup‐0003‐dataS3.zip. [file NPR2-46-e70113-s001.zip › Event Related Raw Tiff/Animal5_42db_ave.tif]

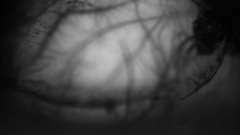

Supplement: Supplementary file 3 — Data S3: npr270113‐sup‐0003‐dataS3.zip. [file NPR2-46-e70113-s001.zip › Event Related Raw Tiff/Animal5_44db_ave.tif]

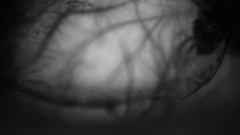

Supplement: Supplementary file 3 — Data S3: npr270113‐sup‐0003‐dataS3.zip. [file NPR2-46-e70113-s001.zip › Event Related Raw Tiff/Animal5_46db_ave.tif]

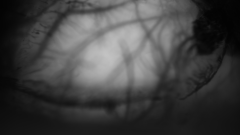

Supplement: Supplementary file 3 — Data S3: npr270113‐sup‐0003‐dataS3.zip. [file NPR2-46-e70113-s001.zip › Event Related Raw Tiff/Animal5_48db_ave.tif]

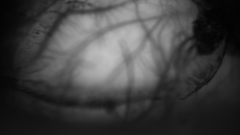

Supplement: Supplementary file 3 — Data S3: npr270113‐sup‐0003‐dataS3.zip. [file NPR2-46-e70113-s001.zip › Event Related Raw Tiff/Animal5_50db_ave.tif]

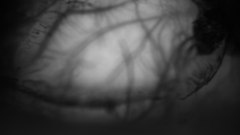

Supplement: Supplementary file 3 — Data S3: npr270113‐sup‐0003‐dataS3.zip. [file NPR2-46-e70113-s001.zip › Event Related Raw Tiff/Animal5_52db_ave.tif]

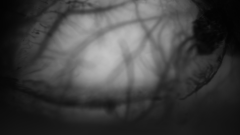

Supplement: Supplementary file 3 — Data S3: npr270113‐sup‐0003‐dataS3.zip. [file NPR2-46-e70113-s001.zip › Event Related Raw Tiff/Animal5_54db_ave.tif]

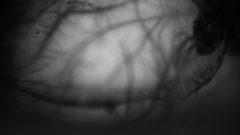

Supplement: Supplementary file 3 — Data S3: npr270113‐sup‐0003‐dataS3.zip. [file NPR2-46-e70113-s001.zip › Event Related Raw Tiff/Animal5_56db_ave.tif]

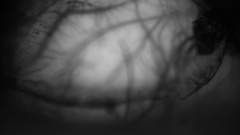

Supplement: Supplementary file 3 — Data S3: npr270113‐sup‐0003‐dataS3.zip. [file NPR2-46-e70113-s001.zip › Event Related Raw Tiff/Animal5_58db_ave.tif]

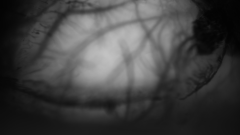

Supplement: Supplementary file 3 — Data S3: npr270113‐sup‐0003‐dataS3.zip. [file NPR2-46-e70113-s001.zip › Event Related Raw Tiff/Animal5_60db_ave.tif]

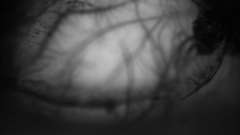

Supplement: Supplementary file 3 — Data S3: npr270113‐sup‐0003‐dataS3.zip. [file NPR2-46-e70113-s001.zip › Event Related Raw Tiff/Animal5_62db_ave.tif]

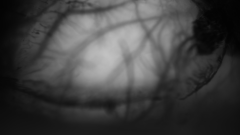

Supplement: Supplementary file 3 — Data S3: npr270113‐sup‐0003‐dataS3.zip. [file NPR2-46-e70113-s001.zip › Event Related Raw Tiff/Animal5_64db_ave.tif]

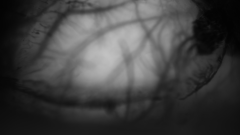

Supplement: Supplementary file 3 — Data S3: npr270113‐sup‐0003‐dataS3.zip. [file NPR2-46-e70113-s001.zip › Event Related Raw Tiff/Animal5_66db_ave.tif]

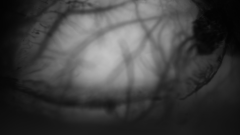

Supplement: Supplementary file 3 — Data S3: npr270113‐sup‐0003‐dataS3.zip. [file NPR2-46-e70113-s001.zip › Event Related Raw Tiff/Animal5_68db_ave.tif]

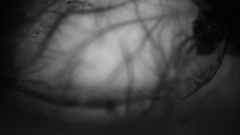

Supplement: Supplementary file 3 — Data S3: npr270113‐sup‐0003‐dataS3.zip. [file NPR2-46-e70113-s001.zip › Event Related Raw Tiff/Animal5_70db_ave.tif]

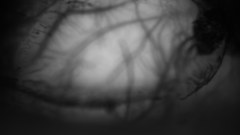

Supplement: Supplementary file 3 — Data S3: npr270113‐sup‐0003‐dataS3.zip. [file NPR2-46-e70113-s001.zip › Event Related Raw Tiff/Animal5_72db_ave.tif]

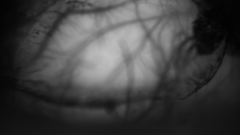

Supplement: Supplementary file 3 — Data S3: npr270113‐sup‐0003‐dataS3.zip. [file NPR2-46-e70113-s001.zip › Event Related Raw Tiff/Animal5_74db_ave.tif]

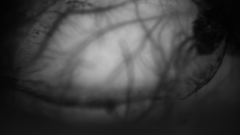

Supplement: Supplementary file 3 — Data S3: npr270113‐sup‐0003‐dataS3.zip. [file NPR2-46-e70113-s001.zip › Event Related Raw Tiff/Animal5_76db_ave.tif]

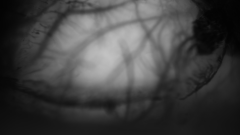

Supplement: Supplementary file 3 — Data S3: npr270113‐sup‐0003‐dataS3.zip. [file NPR2-46-e70113-s001.zip › Event Related Raw Tiff/Animal5_78db_ave.tif]

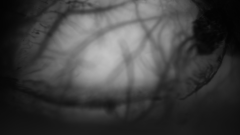

Supplement: Supplementary file 3 — Data S3: npr270113‐sup‐0003‐dataS3.zip. [file NPR2-46-e70113-s001.zip › Event Related Raw Tiff/Animal5_80db_ave.tif]

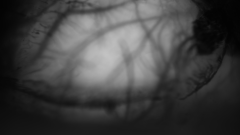

Supplement: Supplementary file 3 — Data S3: npr270113‐sup‐0003‐dataS3.zip. [file NPR2-46-e70113-s001.zip › Event Related Raw Tiff/Animal5_82db_ave.tif]

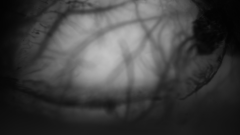

Supplement: Supplementary file 3 — Data S3: npr270113‐sup‐0003‐dataS3.zip. [file NPR2-46-e70113-s001.zip › Event Related Raw Tiff/Animal5_84db_ave.tif]

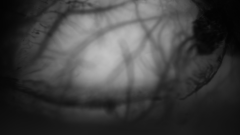

Supplement: Supplementary file 3 — Data S3: npr270113‐sup‐0003‐dataS3.zip. [file NPR2-46-e70113-s001.zip › Event Related Raw Tiff/Animal5_86db_ave.tif]

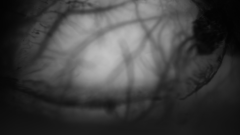

Supplement: Supplementary file 3 — Data S3: npr270113‐sup‐0003‐dataS3.zip. [file NPR2-46-e70113-s001.zip › Event Related Raw Tiff/Animal5_88db_ave.tif]

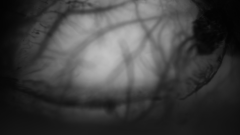

Supplement: Supplementary file 3 — Data S3: npr270113‐sup‐0003‐dataS3.zip. [file NPR2-46-e70113-s001.zip › Event Related Raw Tiff/Animal5_90db_ave.tif]

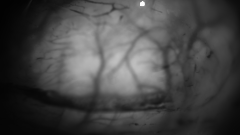

Supplement: Supplementary file 3 — Data S3: npr270113‐sup‐0003‐dataS3.zip. [file NPR2-46-e70113-s001.zip › Event Related Raw Tiff/Animal6_42db_ave.tif]

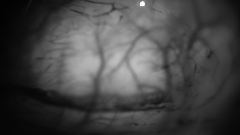

Supplement: Supplementary file 3 — Data S3: npr270113‐sup‐0003‐dataS3.zip. [file NPR2-46-e70113-s001.zip › Event Related Raw Tiff/Animal6_44db_ave.tif]

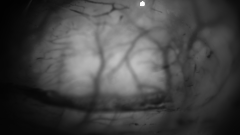

Supplement: Supplementary file 3 — Data S3: npr270113‐sup‐0003‐dataS3.zip. [file NPR2-46-e70113-s001.zip › Event Related Raw Tiff/Animal6_46db_ave.tif]

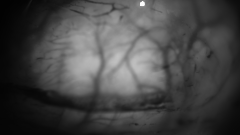

Supplement: Supplementary file 3 — Data S3: npr270113‐sup‐0003‐dataS3.zip. [file NPR2-46-e70113-s001.zip › Event Related Raw Tiff/Animal6_48db_ave.tif]

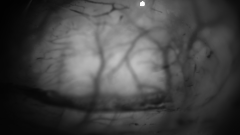

Supplement: Supplementary file 3 — Data S3: npr270113‐sup‐0003‐dataS3.zip. [file NPR2-46-e70113-s001.zip › Event Related Raw Tiff/Animal6_50db_ave.tif]

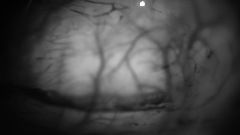

Supplement: Supplementary file 3 — Data S3: npr270113‐sup‐0003‐dataS3.zip. [file NPR2-46-e70113-s001.zip › Event Related Raw Tiff/Animal6_52db_ave.tif]

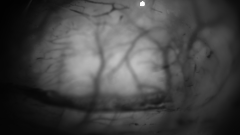

Supplement: Supplementary file 3 — Data S3: npr270113‐sup‐0003‐dataS3.zip. [file NPR2-46-e70113-s001.zip › Event Related Raw Tiff/Animal6_54db_ave.tif]

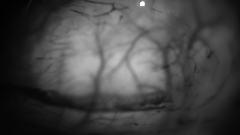

Supplement: Supplementary file 3 — Data S3: npr270113‐sup‐0003‐dataS3.zip. [file NPR2-46-e70113-s001.zip › Event Related Raw Tiff/Animal6_56db_ave.tif]

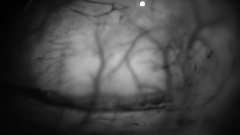

Supplement: Supplementary file 3 — Data S3: npr270113‐sup‐0003‐dataS3.zip. [file NPR2-46-e70113-s001.zip › Event Related Raw Tiff/Animal6_58db_ave.tif]

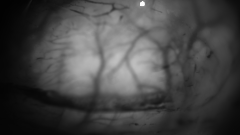

Supplement: Supplementary file 3 — Data S3: npr270113‐sup‐0003‐dataS3.zip. [file NPR2-46-e70113-s001.zip › Event Related Raw Tiff/Animal6_60db_ave.tif]

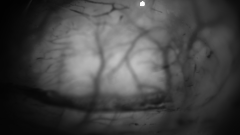

Supplement: Supplementary file 3 — Data S3: npr270113‐sup‐0003‐dataS3.zip. [file NPR2-46-e70113-s001.zip › Event Related Raw Tiff/Animal6_62db_ave.tif]

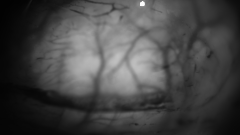

Supplement: Supplementary file 3 — Data S3: npr270113‐sup‐0003‐dataS3.zip. [file NPR2-46-e70113-s001.zip › Event Related Raw Tiff/Animal6_64db_ave.tif]

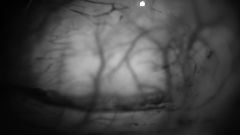

Supplement: Supplementary file 3 — Data S3: npr270113‐sup‐0003‐dataS3.zip. [file NPR2-46-e70113-s001.zip › Event Related Raw Tiff/Animal6_66db_ave.tif]

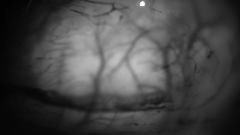

Supplement: Supplementary file 3 — Data S3: npr270113‐sup‐0003‐dataS3.zip. [file NPR2-46-e70113-s001.zip › Event Related Raw Tiff/Animal6_68db_ave.tif]

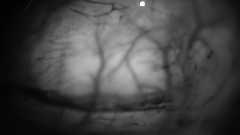

Supplement: Supplementary file 3 — Data S3: npr270113‐sup‐0003‐dataS3.zip. [file NPR2-46-e70113-s001.zip › Event Related Raw Tiff/Animal6_70db_ave.tif]

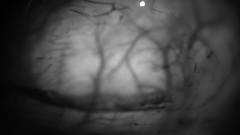

Supplement: Supplementary file 3 — Data S3: npr270113‐sup‐0003‐dataS3.zip. [file NPR2-46-e70113-s001.zip › Event Related Raw Tiff/Animal6_72db_ave.tif]

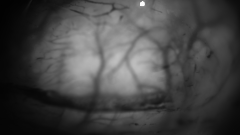

Supplement: Supplementary file 3 — Data S3: npr270113‐sup‐0003‐dataS3.zip. [file NPR2-46-e70113-s001.zip › Event Related Raw Tiff/Animal6_74db_ave.tif]

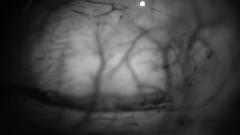

Supplement: Supplementary file 3 — Data S3: npr270113‐sup‐0003‐dataS3.zip. [file NPR2-46-e70113-s001.zip › Event Related Raw Tiff/Animal6_76db_ave.tif]

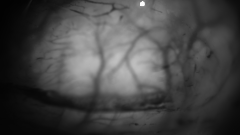

Supplement: Supplementary file 3 — Data S3: npr270113‐sup‐0003‐dataS3.zip. [file NPR2-46-e70113-s001.zip › Event Related Raw Tiff/Animal6_78db_ave.tif]

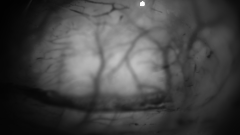

Supplement: Supplementary file 3 — Data S3: npr270113‐sup‐0003‐dataS3.zip. [file NPR2-46-e70113-s001.zip › Event Related Raw Tiff/Animal6_80db_ave.tif]

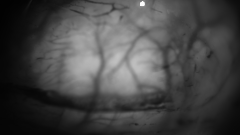

Supplement: Supplementary file 3 — Data S3: npr270113‐sup‐0003‐dataS3.zip. [file NPR2-46-e70113-s001.zip › Event Related Raw Tiff/Animal6_82db_ave.tif]

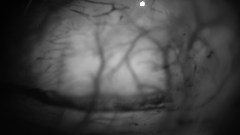

Supplement: Supplementary file 3 — Data S3: npr270113‐sup‐0003‐dataS3.zip. [file NPR2-46-e70113-s001.zip › Event Related Raw Tiff/Animal6_84db_ave.tif]

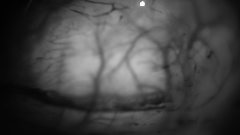

Supplement: Supplementary file 3 — Data S3: npr270113‐sup‐0003‐dataS3.zip. [file NPR2-46-e70113-s001.zip › Event Related Raw Tiff/Animal6_86db_ave.tif]

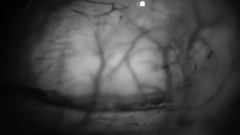

Supplement: Supplementary file 3 — Data S3: npr270113‐sup‐0003‐dataS3.zip. [file NPR2-46-e70113-s001.zip › Event Related Raw Tiff/Animal6_88db_ave.tif]

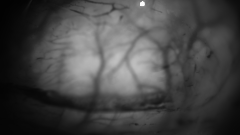

Supplement: Supplementary file 3 — Data S3: npr270113‐sup‐0003‐dataS3.zip. [file NPR2-46-e70113-s001.zip › Event Related Raw Tiff/Animal6_90db_ave.tif]
